# Regulated Erlin-dependent release of the B12 transmembrane J-protein promotes ER membrane penetration of a non-enveloped virus

**DOI:** 10.1371/journal.ppat.1006439

**Published:** 2017-06-14

**Authors:** Takamasa Inoue, Billy Tsai

**Affiliations:** Department of Cell and Developmental Biology, University of Michigan Medical School, Ann Arbor, MI, United States of America; Brown University, UNITED STATES

## Abstract

The molecular mechanism by which non-enveloped viruses penetrate biological membranes remains enigmatic. The non-enveloped polyomavirus SV40 penetrates the endoplasmic reticulum (ER) membrane to reach the cytosol and cause infection. We previously demonstrated that SV40 creates its own membrane penetration structure by mobilizing select transmembrane proteins to distinct puncta in the ER membrane called foci that likely function as the cytosol entry sites. How these ER membrane proteins reorganize into the foci is unknown. B12 is a transmembrane J-protein that mobilizes into the foci to promote cytosol entry of SV40. Here we identify two closely related ER membrane proteins Erlin1 and Erlin2 (Erlin1/2) as B12-interaction partners. Strikingly, SV40 recruits B12 to the foci by inducing release of this J-protein from Erlin1/2. Our data thus reveal how a non-enveloped virus promotes its own membrane translocation by triggering the release and recruitment of a critical transport factor to the membrane penetration site.

## Author summary

Polyomavirus (PyV) is a non-enveloped DNA tumor virus that causes debilitating human diseases especially in immunocompromised individuals. At the cellular level, PyVs such as the simian PyV SV40 must enter a host cell and penetrate the ER membrane to reach the cytosol in order to cause infection. Prior to ER membrane transport, SV40 reorganizes select ER membrane proteins including the J-protein B12 to potential membrane penetration sites on the ER membrane called foci where B12 facilitates virus extraction into the cytosol. How B12 reorganizes into the foci is unclear. Here we find that two closely related ER membrane proteins Erlin1 and Erlin2 (Erlin1/2) bind to B12. During infection, SV40 induces release of this J-protein from Erlin1/2 to enable B12 to reorganize into the foci. Our data reveal how a non-enveloped virus mobilizes a specific ER membrane component to a membrane penetration structure to promote its own membrane transport.

## Introduction

Membrane penetration represents a decisive event during virus infection. For enveloped viruses, fusion between the viral and host membranes delivers the core viral particle into the host cytosol [1,2]. By contrast, because a non-enveloped virus lacks a surrounding lipid bilayer, its membrane transport process must be distinct from an enveloped virus. Indeed, membrane penetration by the non-enveloped virus families is not fully understood to date [1–3]. A central enigma that challenges this field is whether a non-enveloped virus hijacks pre-existing channels in the limiting membrane in order to enter the host, or if it generates a membrane transport portal *de novo* and subsequently crosses this structure to reach the host cytosol. Intriguingly, recent reports suggest that the non-enveloped polyomavirus (PyV) creates its own membrane transport structure to enter the host cell and cause infection [4–6], although aspects of this process remain to be clarified.

PyV is a non-enveloped DNA tumor virus known to cause debilitating human diseases especially in immunocompromised individuals. For instance, the human JC PyV is responsible for the fatal demyelinating central nervous system disease progressive multifocal leukoencephalopathy, the BK PyV for BK-associated nephropathy and hemorrhagic cystitis, and the Merkel cell PyV for the aggressive skin cancer Merkel cell carcinoma [7,8]. Structurally, a PyV particle is composed of 72 pentamers of the coat protein VP1, with each pentamer harboring either the internal hydrophobic protein VP2 or VP3 [9–11]. The VP1 pentamers form the outer shell of the virus which in turn encases the 5 kilobase viral DNA genome. A complex network of disulfide bonds, in concert with VP1 C-terminal arms emanating from a pentamer that invade neighboring VP1 pentamers, stabilize the overall viral architecture [10,11]. Because simian PyV SV40 displays both structural and genetic similarities to human PyVs such as JC and BK PyVs, studies of the SV40 infection pathway have historically illuminated human PyV infection pathways.

To cause infection at the cellular level, SV40 binds to the ganglioside GM1 receptor on the plasma membrane [12,13], entering the host cell via receptor-mediated endocytosis. Upon entry, the virus is initially sorted to endolyososomes [14,15] before being routed to the endoplasmic reticulum (ER) [13,16,17]. Here the virus undergoes conformational changes imparted by ER-resident protein disulfide isomerase (PDI) family members [18,19]. These reactions expose VP2 and VP3, generating a hydrophobic particle that physically binds to and integrates into the ER membrane [5]. The membrane-embedded virus is initially recognized by the ER membrane protein BAP31 [5], transiently stabilized by the transmembrane protein EMC1 [20], and eventually ejected into the cytosol by the Hsc70-SGTA-Hsp105 cytosolic extraction machinery [6,21]. This machinery is associated with the cytosolic side of the ER membrane via interaction with three different ER membrane J-proteins called DNAJB12 (B12), DNAJB14 (B14) and DNAJC18 (C18) [4,6,21,22]. A J-protein typically binds to Hsp70 family proteins and stimulates their ATPase activity, enabling the Hsp70 proteins to efficiently capture their clients [23]. From the cytosol, the virus is further transported to the nucleus to cause lytic infection or cell transformation.

Strikingly, prior to ER-to-cytosol membrane penetration, SV40 dynamically recruits B12, B14, and C18 (as well as other membrane components) into discrete puncta in the ER membrane called foci [4,6,21]. The kinesin-1 motor promotes maturation of the foci [24]. Because the foci structures are postulated to function as the viral cytosol entry sites [5,6,21], SV40-triggered foci formation represents the first example of a non-enveloped virus creating its own membrane transport portal. What remains a major conundrum is how SV40 induces the reorganization of these membrane J-proteins into the foci.

Using an unbiased proteomics approach, we identify two closely related ER membrane proteins Erlin1 and Erlin2 (Erlin1/2), which preferentially localize in detergent-resistant membranes and hetero-oligomerize to form a massive protein complex [25–28], as B12-interacting partners. Erlin1/2 support SV40-induced foci formation, and promote virus ER-to-cytosol transport, and infection. However, Erlin1/2 themselves do not mobilize into the foci. Instead, our results indicate that these ER membrane proteins bind to and likely anchor B12 in the ER, releasing B12 into the foci upon SV40 infection. Our study thus pinpoints Erlin1/2-dependent release as a key regulatory event enabling B12 to efficiently reorganize into the foci, where this J-protein facilitates virus ER membrane penetration leading to successful infection.

## Results

### B12 binds to the ER membrane proteins Erlin1 and Erlin2

**Fig 1:**
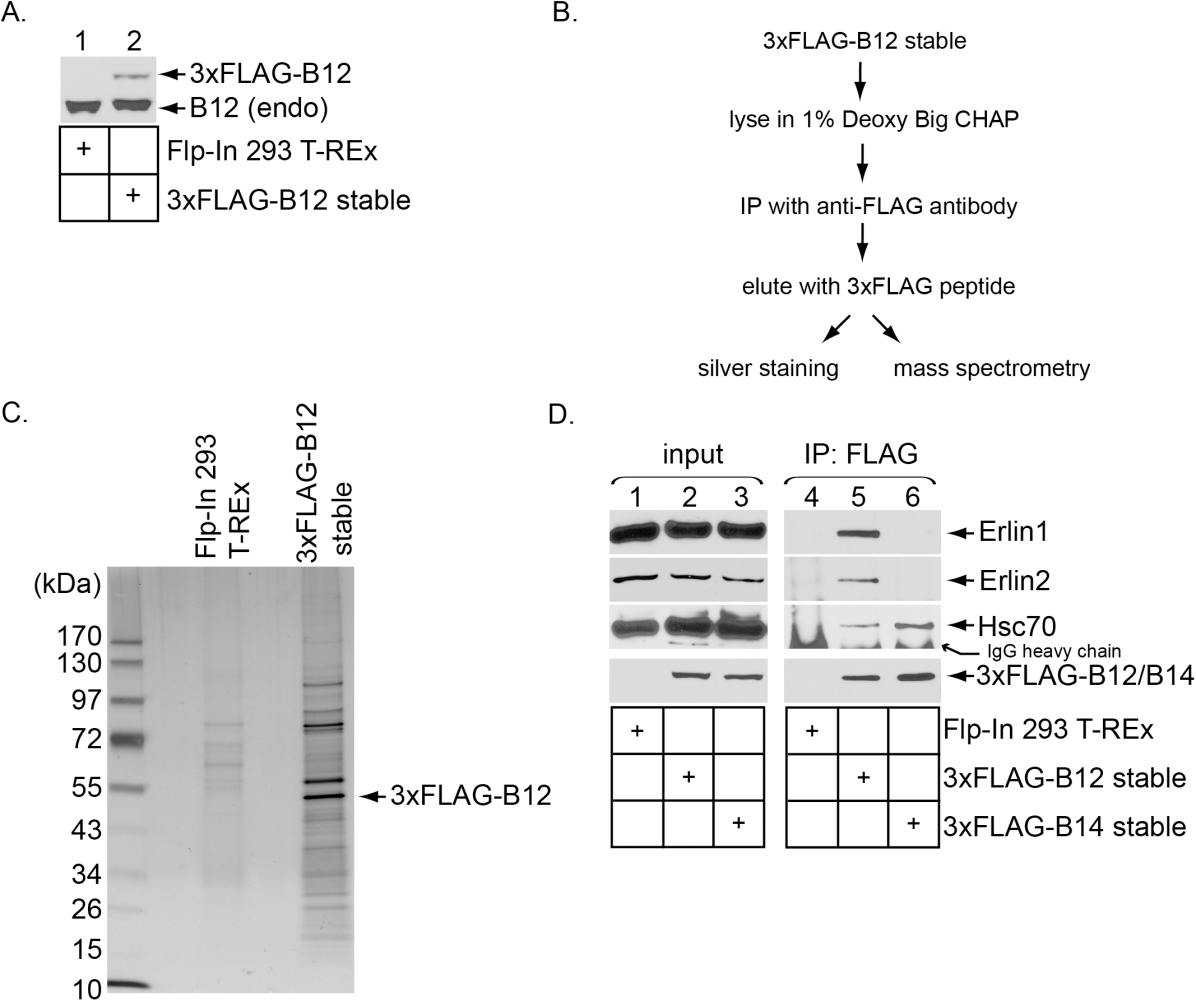
B12 binds to the ER membrane proteins Erlin1 and Erlin2. (A) Parental Flp-In 293 T-REx cells and cell lines stably expressing 3xFLAG-B12 were lysed and the resulting whole cell extracts subjected to SDS-PAGE followed by immunoblotting with an antibody against B12. (B) Strategy used to identify B12-interacting partners. (C) Cell lysates derived from the indicated cell lines were subjected to affinity purification as depicted in B, and the precipitated material analyzed with SDS-PAGE followed by silver staining. (D) Cell lysates derived from the indicated cell lines were subjected to FLAG immunoprecipitation, and the precipitated samples eluted with SDS sample buffer and analyzed by immunoblotting with the indicated antibodies.

Three closely related ER membrane J-proteins called B12, B14 and C18 play critical yet non-overlapping functions in promoting ER-to-cytosol transport and infection of SV40 [4]. We previously identified novel B14- and C18-binding factors that execute distinct functions in supporting this viral membrane transport process [6,20,21]. However, potential B12-interacting proteins that may also play roles during SV40 ER membrane penetration have yet to be identified. To identify possible B12-interacting components, we used Flp-in 293 T-REx cells and generated a cell line stably expressing B12 that is tagged with 3xFLAG at the amino-terminus (3xFLAG-B12 stable); the parental Flp-in 293 T-REx cells not expressing 3xFLAG-B12 were used as the control. When cell extracts derived from the parental Flp-in 293 T-REx and 3xFLAG-B12 stable cells were analyzed by SDS-PAGE, immunoblotting revealed that a lower level of 3xFLAG-B12 was expressed when compared to the endogenous B12 level (Fig 1A, compare lanes 1 and 2). The cell extracts were then subjected to FLAG affinity purification using the schematic depicted in Fig 1B. The FLAG peptide-eluted materials were either analyzed by silver staining or subjected to “shot gun” mass spectrometry. By silver staining, we found the appearance of many distinct bands in the sample derived from the 3xFLAG-B12-expressing cells, while fewer bands of lesser intensity were observed in the sample derived from the parental control cell (Fig 1C). By mass spectrometry, peptides corresponding to more than 1,500 proteins were identified in samples derived from the 3xFLAG-B12 cells, but with only 58 of these proteins having at least 20 peptides (Table 1).

**Table 1:** A list of proteins identified with more than 20 peptides from the 3xFLAG-B12 affinity purified sample.

	total peptides	coverage (%)	gene	Mw (KDa)		total peptides	coverage (%)	gene	Mw (KDa)
1	194	57.12	HSPA8	70.85	31	28	50.68	TUBB4A	49.55
2	134	51.91	TUBB2A	49.87	32	27	23.87	EMC1	111.69
3	115	51.49	VCP	89.27	33	27	35.33	ATAD3A	71.32
4	102	49.22	TUBA1A	50.1	34	27	28.79	EEF1A1	50.11
5	94	49.1	CANX	67.53	35	26	14.83	MAP1B	270.47
6	81	62.19	ATP5B	56.52	36	26	11.39	COPB2	102.42
7	75	53.39	ERLIN2	37.82	37	26	27.37	ATP2A1	110.18
8	69	30.11	HSPA1L	70.33	38	24	21.89	PARP1	113.01
9	64	21.6	HSPA5	72.29	39	24	23.08	HM13	41.46
10	53	49.77	HSPA1A	70.01	40	24	42.18	TUBB	49.64
11	50	12.72	PRKDC	468.79	41	23	32.85	CLCC1	61.98
12	50	40.69	ATP5A1	59.71	42	23	60.61	CDK1	34.07
13	46	43.59	HSPA9	73.63	43	23	36.95	TUFM	49.51
14	46	54.37	RPN1	68.53	44	23	30.92	ERLIN1	38.9
15	46	27.76	IRS4	133.68	45	23	36.91	SLC25A4	33.04
16	45	24.92	SGTA	34.04	46	22	9.58	ITPR3	303.91
17	44	34.89	TUBB3	50.4	47	22	26.75	AIFM1	66.86
18	43	28.45	SLC25A3	40.07	48	22	25.41	PTPLAD1	43.13
19	42	28.69	ATP2A2	114.68	49	22	20.5	HSPA2	69.98
20	42	52.9	STK38	54.16	50	21	24.34	COPB1	107.07
21	41	26.33	NONO	54.2	51	21	21.6	ATP1A1	112.82
22	38	19.55	CAD	242.83	52	21	36.63	UBAC2	38.94
23	38	9.53	TUBB1	50.29	53	21	37.28	DDOST	50.77
24	34	33.74	BCAP31	27.97	54	21	25.89	TRIM21	54.14
25	32	13.03	GCN1L1	292.57	55	21	0.66	RFX7	146.8
27	32	18.23	NUP210	204.98	56	20	23.01	ESYT1	122.78
27	32	29	COPA	138.26	57	20	27.71	SEL1L	88.7
28	32	27.75	NCL	76.57	58	20	42.18	BAG2	23.76
29	32	38.19	RPN2	69.24					
30	31	7.27	MDN1	632.42					

Of the 58 proteins, the ER membrane proteins BAP31, EMC1, and Sel1L were present in this list. Importantly, these proteins were previously demonstrated to participate in ER-to-cytosol membrane penetration of SV40 [5,19,20]. Also included in this list are Erlin1 and Erlin2 (Erlin1/2), two closely related ER membrane proteins that hetero-oligomerize to form a massive protein complex [25,26,28]. Because Erlin1/2 have been implicated in an ER-to-cytosol transport-dependent protein quality control process called ER-associated degradation [27,29,30], we decided to focus on these proteins since SV40 similarly undergoes ER-to-cytosol transport.

To validate the mass spectrometry data and to assess the specificity of the B12-Erlin1/2 interactions, extracts derived from cells stably expressing 3xFLAG-B12 or 3xFLAG-B14, along with the parental cells, were subjected to immunoprecipitation using FLAG antibody-conjugated agarose beads. The precipitated material was subjected to SDS-PAGE followed by immunoblotting. Using this approach, we found that precipitating 3xFLAG-B12 but not 3xFLAG-B14 pulled down endogenous Erlin1/2 (Fig 1D, first and second panels, compare lane 5 to 6). As expected, Hsc70 co-precipitated with 3xFLAG-B12 and 3xFLAG-B14 (Fig 1D, third panel, lanes 5 and 6). These results demonstrate that Erlin1/2 interact specifically with 3xFLAG-B12, in agreement with the mass spectrometry data.

### Erlin1 and Erlin2 execute critical functions during SV40 infection

**Fig 2:**
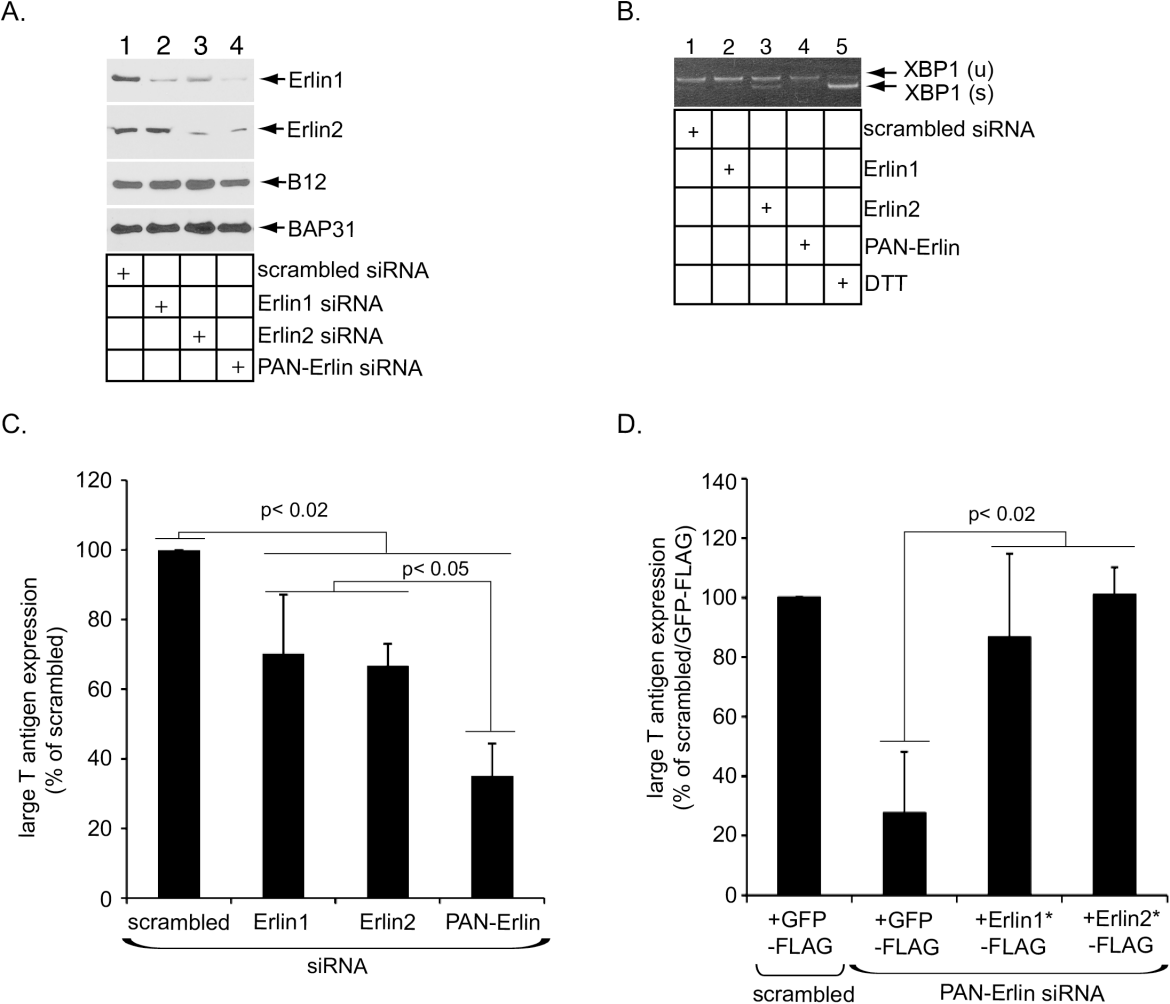
Erlin1 and Erlin2 execute critical functions during SV40 infection. (A) CV-1 cells were transfected with the indicated siRNAs, fractionated with digitonin, and the resulting membrane fractions subjected to SDS-PAGE, followed by immunoblotting with the indicated antibodies. (B) Reverse transcription-PCR (RT-PCR) analysis of the unspliced (u) and spliced (s) forms of the XBP1 mRNA derived from CV-1 cells transfected with the indicated siRNA or treated with DTT. (C) Large T antigen-positive nuclei were scored in SV40-infected CV-1 cells transfected with the indicated siRNAs. Data are normalized against the scrambled siRNA, and represent the means ± standard deviations of data from at least 3 independent experiments. Student two-tailed *t* test was used to determine statistical significance. (D) CV-1 cells initially transfected with scrambled or PAN-Erlin siRNA were subsequently transfected with GFP-FLAG, Erlin1*-FLAG, or Erlin2*-FLAG. Cells were then infected with SV40 for 24 h and subjected to immunofluorescence analyses using antibodies against FLAG and large T antigen. Only cells expressing the indicated FLAG protein were scored for large T antigen expression. Data are normalized against cells that were initially transfected with scrambled siRNA and subsequently transfected with GFP-FLAG, and represent the means ± standard deviations of data from at least 3 independent experiments. Student two-tailed *t* test was used to determine statistical significance.

We next examined if Erlin1/2 play roles during SV40 infection. Simian CV-1 cells, which are typically used to study SV40 infection, were transfected with siRNA targeting either Erlin1, Erlin2, or both (PAN-Erlin). The resulting cell extracts were subjected to SDS-PAGE followed by immunoblotting with antibodies against Erlin1 and Erlin2. We found that Erlin1 siRNA downregulated Erlin1 expression (Fig 2A, first panel, compare lane 2 to 1) without affecting Erlin2 expression (Fig 2A, second panel, compare lane 2 to 1). Erlin2 siRNA silenced Erlin2 expression (Fig 2A, second panel, compare lane 3 to 1) while also moderately reducing Erlin1 expression (Fig 2A, top panel, compare lane 3 to lane 1), suggesting that this siRNA may target Erlin1 to some extent. As expected, the PAN-Erlin siRNA decreased both Erlin1 and Erlin2 levels (Fig 2A, first and second panels, compare lane 4 to 1). To determine if severe ER stress is induced upon depletion of the Erlins, we monitored splicing of the XBP1 mRNA by RT-PCR analysis. XBP1 splicing typically ensues when misfolded proteins massively accumulate in the ER, and is considered the most sensitive indicator of ER stress induction [31]. We found that when compared to cells treated with the chemical ER stress inducer dithiothreitol (DTT), neither the Erlin1, Erlin2, nor PAN-Erlin siRNA triggered significant ER stress (Fig 2B, compare lane 5 to lanes 1–4).

Under these conditions, we assessed SV40 infection by scoring cells for expression of the virally encoded large T antigen, a hallmark for SV40 infection, in the host nucleus using an immunofluorescence-based approach. While depleting either Erlin1 or Erlin2 individually blocked infection moderately (Fig 2C, compare second and third to first bar), simultaneous knockdown of Erlin1/2 potently reduced SV40 infection (Fig 2C, compare fourth to first bar). To verify that this decreased infection is indeed caused by the knockdown of Erlin1 and Erlin2 and not due to unexpected off-target effects, we employed a rescue experiment by transfecting siRNA-resistant FLAG-tagged Erlin1 (Erlin1*-FLAG), Erlin2 (Erlin2*-FLAG), or the control GFP-FLAG construct in cells simultaneously depleted of Erlin1/2. Only cells expressing the FLAG protein were scored. Using this approach, we found that add-back of either Erlin1*-FLAG or Erlin2*-FLAG fully restored the block in SV40 infection imposed by Erlin1/2 depletion (Fig 2D, compare third and fourth to first and second bars). These findings demonstrate that the robust decrease in SV40 infection due to the PAN-Erlin1/2 siRNA results from silencing Erlin1/2, thus unambiguously revealing a critical function of Erlin1/2 in SV40 infection. Importantly, as reconstituting either Erlin1 or Erlin2 fully restored the infection block caused by silencing Erlin1/2, these related membrane proteins likely execute overlapping functions during SV40 infection.

### Erlin1 and Erlin2 support SV40 ER-to-cytosol transport and virus-induced foci formation

**Fig 3:**
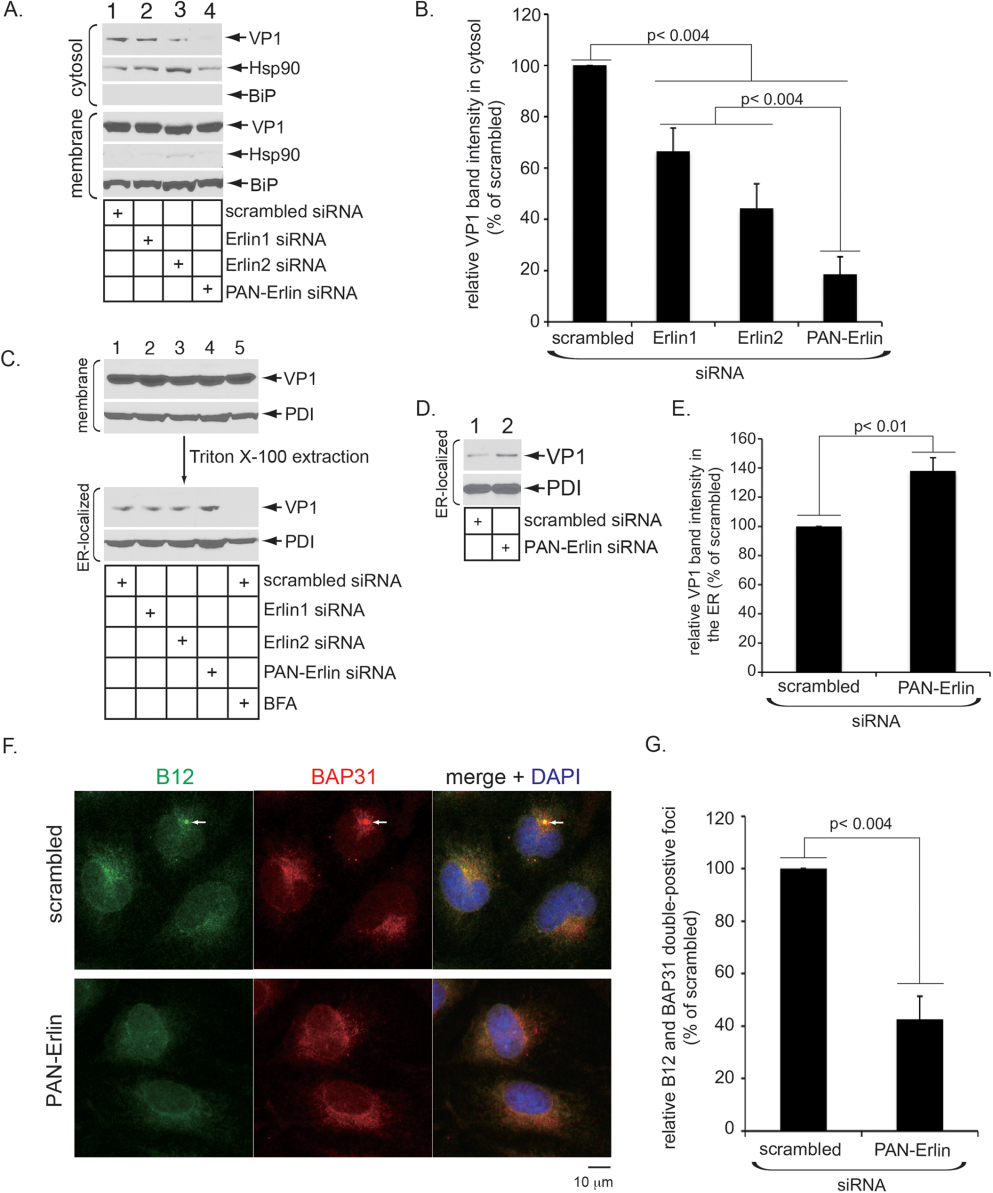
Erlin1 and Erlin2 support SV40 ER-to-cytosol transport and virus-induced foci formation. (A) CV-1 cells transfected with the indicated siRNA were infected with SV40 for 12 h, harvested, treated with digitonin, and centrifuged. The resulting supernatant fraction containing cytosolic proteins (cytosol) and the pellet fraction containing membranous organelles (membrane) were analyzed by SDS-PAGE followed by immunoblotting with the indicated antibodies. (B) The VP1 band intensity in the cytosol fraction in A was quantified using ImageJ (NIH). Data were normalized against cells transfected with scrambled siRNA, and represent the means ± standard deviations of data from at least 3 independent experiments. Student two-tailed *t* test was used to determine statistical significance. (C) The membrane fraction in A was further solubilized with Triton X-100 and centrifuged. The resulting supernatant fractions (Triton X-100 soluble membrane) containing ER-localized SV40 were analyzed by immunoblotting with the indicated antibodies. (D) CV-1 cells transfected with the scrambled or PAN-Erlin siRNA were infected with SV40 for 12 h, and subjected to a fractionation approach using digitonin as in A. The membrane fraction was further solubilized with Triton X-100, and centrifuged as in C. The resulting supernatant fraction was analyzed by immunoblotting with the indicated antibodies. (E) The VP1 band intensity of ER-localized SV40 in cells transfected with scrambled or PAN-Erlin siRNA in C and D was quantified using ImageJ (NIH). Data were normalized against cells transfected with the scrambled siRNA, and represent the means ± standard deviations of data from at least 3 independent experiments. Student two-tailed *t* test was used to determine statistical significance. (F) CV-1 cells transfected with scrambled or PAN-Erlin siRNA were infected with SV40 for 8 h, fixed, and subjected to immunofluorescence staining with antibodies against B12 and BAP31, and DAPI (4, 6-diamidino-2-phenylindole) staining. Images were taken by epifluorescence microscopy. Arrows indicate SV40-induced foci. Bar represents 10 μm. (G) Numbers of cells with at least one B12 and BAP31 double-positive focus in SV40-infected cells transfected with the indicated siRNA were counted. Data are normalized against cells transfected with the scrambled siRNA, and represent the means ± standard deviations of data from at least 3 independent experiments. Student two-tailed *t* test was used to determine statistical significance.

Because ER-to-cytosol transport is essential for successful SV40 infection, we asked if Erlin1/2 promote this membrane transport process using a cell-based, semi-permeabilized transport assay that we previously developed [32]. Briefly, cells are treated with a low concentration of digitonin that selectively permeabilizes the plasma membrane without damaging internal membranes. The sample is then centrifuged to generate two fractions, a supernatant fraction that contains cytosolic proteins and SV40 that has penetrated the ER membrane to reach the cytosol (cytosol), and a pellet fraction that contains membranous organelles, including the ER, as well as any ER-localized virus (membrane). Cells transfected with scrambled, Erlin1, Erlin2 or PAN-Erlin siRNA were infected with SV40, and subjected to this fractionation procedure, with the resulting cytosol and membrane fractions analyzed by immunoblotting. We found that while the majority of the cytosolic marker Hsp90 was detected in the cytosol fraction (Fig 3A, compare second to fifth panel), the ER marker BiP was found only in the membrane fraction (Fig 3A, compare sixth to third panel), thus verifying the integrity of the fractionation procedure. Importantly, under this condition, we found that although silencing either Erlin1 or Erlin2 moderately decreased the SV40 VP1 level in the cytosol fraction (Fig 3A, top panel, compare lanes 2 and 3 to 1; the VP1 level is quantified in Fig 3B), simultaneous knockdown of Erlin1/2 potently impaired VP1 appearance in the cytosol fraction (Fig 3A, top panel, compare lane 4 to 1; quantified in Fig 3B). These findings strongly suggest that Erlin1/2 support SV40’s ER membrane transport event.

We next used a biochemical fractionation protocol based on Triton X-100 extraction to isolate ER-localized SV40 from the membrane fraction [32] (see also Materials and Methods). We found that the level of ER-localized SV40 did not decrease by depleting Erlin1, Erlin2, or both Erlin1/2 (Fig 3C, third panel, compare lanes 2–4 to 1), whereas it did significantly decrease when cells were treated with brefeldin A (BFA) which blocks SV40 trafficking from the plasma membrane to the ER (Fig 3C, third panel, compare lane 5 to 1), as expected. In fact, knockdown of Erlin1/2 moderately increased the level of ER-localized SV40 when compared to the control condition (Fig 3C, third panel, compare lane 4 to 1), suggesting SV40 is trapped in the ER in the absence of Erlin1/2. This modest increase was again evident when a lesser amount of the samples was analyzed and quantified (Fig 3D, top panel, compare lane 2 to 1; quantified in Fig 3E). Because only a small fraction of total ER-localized SV40 reaches the cytosol (see Fig 3A, compare VP1 level in first versus fourth panels), the virus level that accumulates in the ER due to a block in cytosol entry is expected to be modest relative to the total amount of virus in the ER. Thus, the decreased VP1 level in the cytosol fractions observed when Erlin1, Erlin2, or Erlin1/2 were depleted is not due to defective ER arrival of SV40 from the cell surface. These data establish Erlin1/2 as being critical for supporting SV40 ER-to-cytosol transport, consistent with the importance of these factors during SV40 infection (Fig 2).

During infection, SV40 triggers mobilization of select ER membrane proteins including B12, B14, C18, and BAP31 into distinct puncta on the ER referred to as foci. The virus-induced foci structures have been proposed to function as SV40’s cytosol entry sites from the ER [4–6,21]. To begin to address the molecular basis by which Erlin1/2 promote ER membrane penetration of SV40, we asked if these ER membrane proteins support foci formation. Accordingly, cells transfected with scrambled or PAN-Erlin siRNA were infected with SV40, and subjected to immunofluorescence using antibodies against B12 and BAP31 to score for appearance of B12 and BAP31 double-positive foci. Our epifluorescence microscopy-based analyses revealed that silencing Erlin1/2 decreased formation of SV40-induced, B12/BAP31-containing foci by approximately 60% (Fig 3F, compare top and bottom panels; the level of B12/BAP31-containing foci was quantified in Fig 3G). We conclude that Erlin1/2 play a pivotal function during SV40-triggered foci formation–this likely explains why Erlin1/2 are crucial for SV40’s ER-to-cytosol membrane transport (Fig 3A and 3B) leading to successful infection (Fig 2).

### Interactions with Erlin1 and Erlin2 are critical for B12 to mobilize into the foci to support SV40 infection

**Fig 4:**
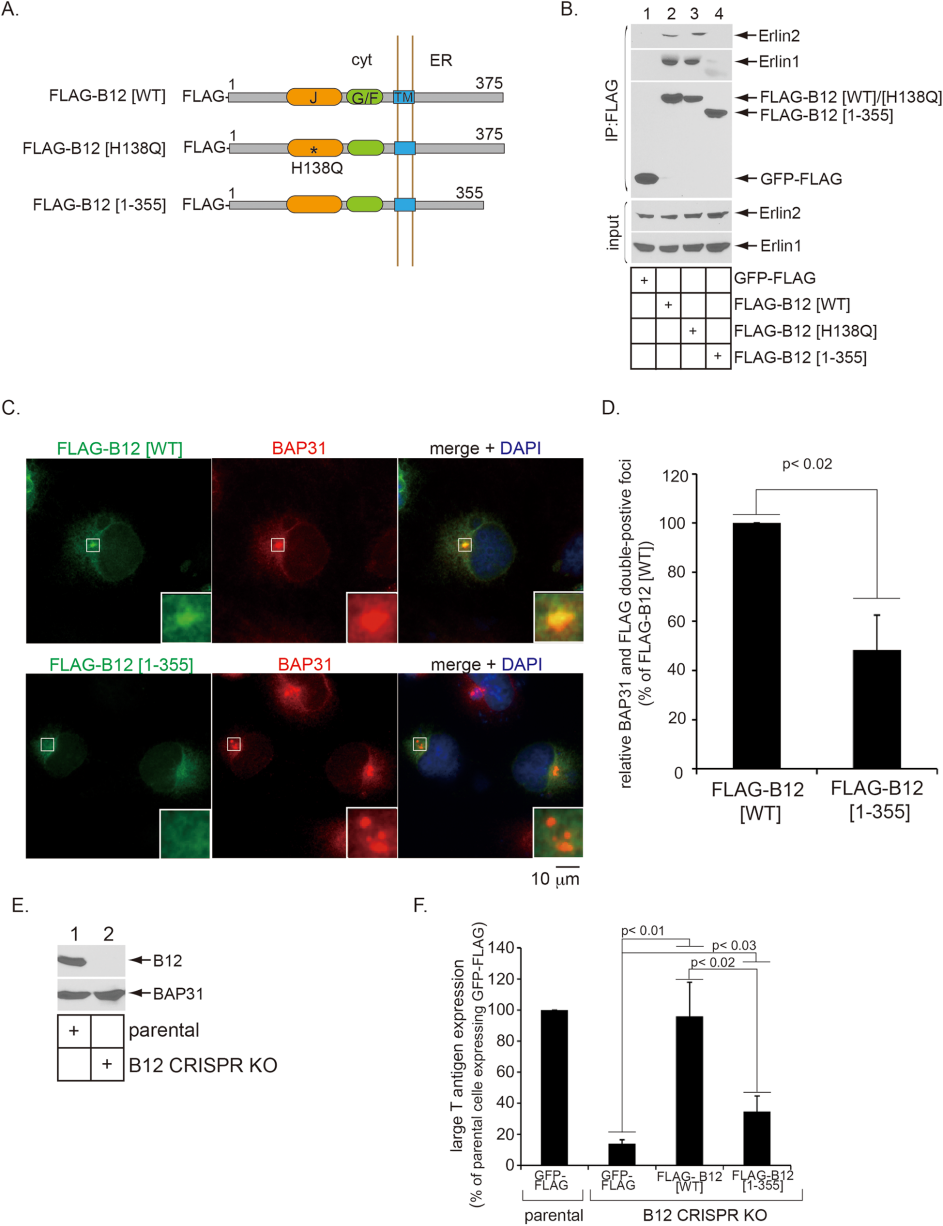
Interactions with Erlin1 and Erlin2 are critical for B12 to mobilize into the foci and support infection. (A) Schematics of the N-terminal FLAG-tagged B12 constructs used in this figure. Wild type B12 (B12 [WT]) is composed of 375 amino acids with three domains: J-domain (J, 112–176 aa), glycine/phenylalanine-rich domain (G/F, 184–213 aa) and transmembrane domain (TM, 244–264 aa). Hsp70-binding defective B12 (B12 [H138Q]) harbors a mutation in which histidine at position 138 is changed to glutamine. Mutant B12 (B12 [1–355]) lacks the last 20 amino acids from the C-terminus. (B) 293T cells transfected with the indicated FLAG-tagged constructs were lysed and the resulting whole cell lysates were subjected to immunoprecipitation using FLAG antibody-conjugated agarose beads. Whole cell lysate (input) and immunoprecipitated material were analyzed by immunoblotting with the indicated antibodies. (C) CV-1 cells transfected with the indicated FLAG-tagged constructs were infected with SV40 for 16 h, fixed, and subjected to the immunofluorescence assay using antibodies against BAP31 and FLAG. Images were taken by epifluorescence microscopy. Inset shows a 3x enlarged image corresponding to the enclosed white square. Bar represents 10 μm. (D) Numbers of cells with at least one BAP31 and FLAG double-positive focus in SV40-infected cells were counted, as in Fig 3E. Data are normalized against cells transfected with FLAG-B12 [WT], and represent the means ± standard deviations of data from at least 3 independent experiments. Student two-tailed *t* test was used to determine statistical significance. (E) Parental and B12 CRISPR KO CV-1 cells were lysed and the resulting whole cell lysates were analyzed by immunoblotting with the indicated antibodies. (F) Parental and B12 CRISPR KO CV-1 cells transfected with the indicated FLAG-tagged constructs were infected with SV40 and subjected to immunofluorescence analysis using antibodies against FLAG and large T antigen. FLAG and large T antigen double-positive cells were counted and analyzed as in Fig 2D. Data are normalized against parental cells transfected with GFP-FLAG, and represent the means ± standard deviations of data from at least 3 independent experiments. Student two-tailed *t* test was used to determine statistical significance.

Since Erlin1/2 were initially identified as B12-interacting partners, we asked if B12’s ability to interact with Erin1/2 enables B12 to mobilize into the foci in order to support viral infection. To evaluate this idea, we first sought to identify a B12 mutant that cannot bind to Erlin1/2. Since Erlin1/2 have a short cytosolic tail followed by single transmembrane domain and an extended ER luminal region, the B12-Erlin1/2 interaction is likely mediated in the ER. In addition, the C-terminal luminal domains of B12 and B14 display a high degree of sequence variations. Because B12 but not B14 interacts with Erlin1/2 (Fig 1D), we reasoned that B12’s C-terminal domain is likely responsible for engaging Erlin1/2. To test this, we constructed WT B12 and a B12 mutant lacking the last 20 amino acids of its C-terminus, with both constructs containing a single FLAG tag appended at the N-terminus (FLAG-B12 [WT] and FLAG-B12 [1–355]; Fig 4A). As control constructs, in addition to GFP-FLAG, we generated a B12 point mutant in which a mutation is introduced within its cytosolic J-domain (FLAG-B12 [H138Q]; Fig 4A); while this mutation renders the protein incapable of binding to cytosolic Hsc70, its luminal C-terminus should remain intact. In cells expressing either GFP-FLAG, FLAG-B12 [WT], FLAG-B12 [H138Q], or FLAG-B12 [1–355], we found that only pull down of FLAG-B12 [WT] or FLAG-B12 [H138Q] but not GFP-FLAG or FLAG-B12 [1–355] co-precipitated endogenous Erlin1/2 (Fig 4B, first and second panels, compare lanes 2 and 3 to lanes 1 and 4). These findings demonstrate that FLAG-B12 [1–355] cannot interact with Erlin1/2, indicating that B12’s last 20 amino acids are required for binding to Erlin1/2. Importantly, in contrast to FLAG-B12 [WT], FLAG-B12 [1–355] inefficiently mobilizes into the BAP31-containing foci during SV40 infection (Fig 4C, compare top to bottom row; the level of BAP31 and FLAG double-positive foci is quantified in Fig 4D). These data suggest that B12’s interactions with Erlin1/2 are important for the efficient mobilization of B12 into the foci during virus infection.

To probe if FLAG-B12 [1–355]’s inability to mobilize into the foci affects virus infection, we generated a B12 CRISPR knockout (KO) cell line (Fig 4E, top panel, compare lane 2 to 1), which was sequenced to confirm the mutation (S1 Fig). SV40 was then incubated with the parental cell line transfected with GFP-FLAG, or the CRISPR B12 KO cell line transfected with either GFP-FLAG, FLAG-B12 [WT], or FLAG-B12 [1–355]. Virus infection was assessed by scoring for the expression of large T antigen in only FLAG-expressing cells. Using this strategy, we found that SV40 infection was potently blocked in the CRISPR B12 KO cell line when compared to the parental control cells (Fig 4F, compare second to first bar), consistent with our previous studies using an RNAi-mediated knockdown strategy [4,22]. Strikingly, addback of FLAG-B12 [WT] but not FLAG-B12 [1–355] into the B12 KO cells fully restored infection (Fig 4F, compare third to fourth bar), indicating that FLAG-B12 [1–355] cannot support SV40 infection. Thus, as a mutant B12 that cannot bind to Erlin1/2 nor efficiently mobilize into the foci also fails to restore infection, these data strongly suggest that B12’s interaction with Erlin1/2 is essential for its reorganization into the foci where B12 facilitates SV40 ER membrane penetration leading to successful infection.

### Erlin1 and Erlin2 do not reorganize into the SV40-induced foci structures

**Fig 5:**
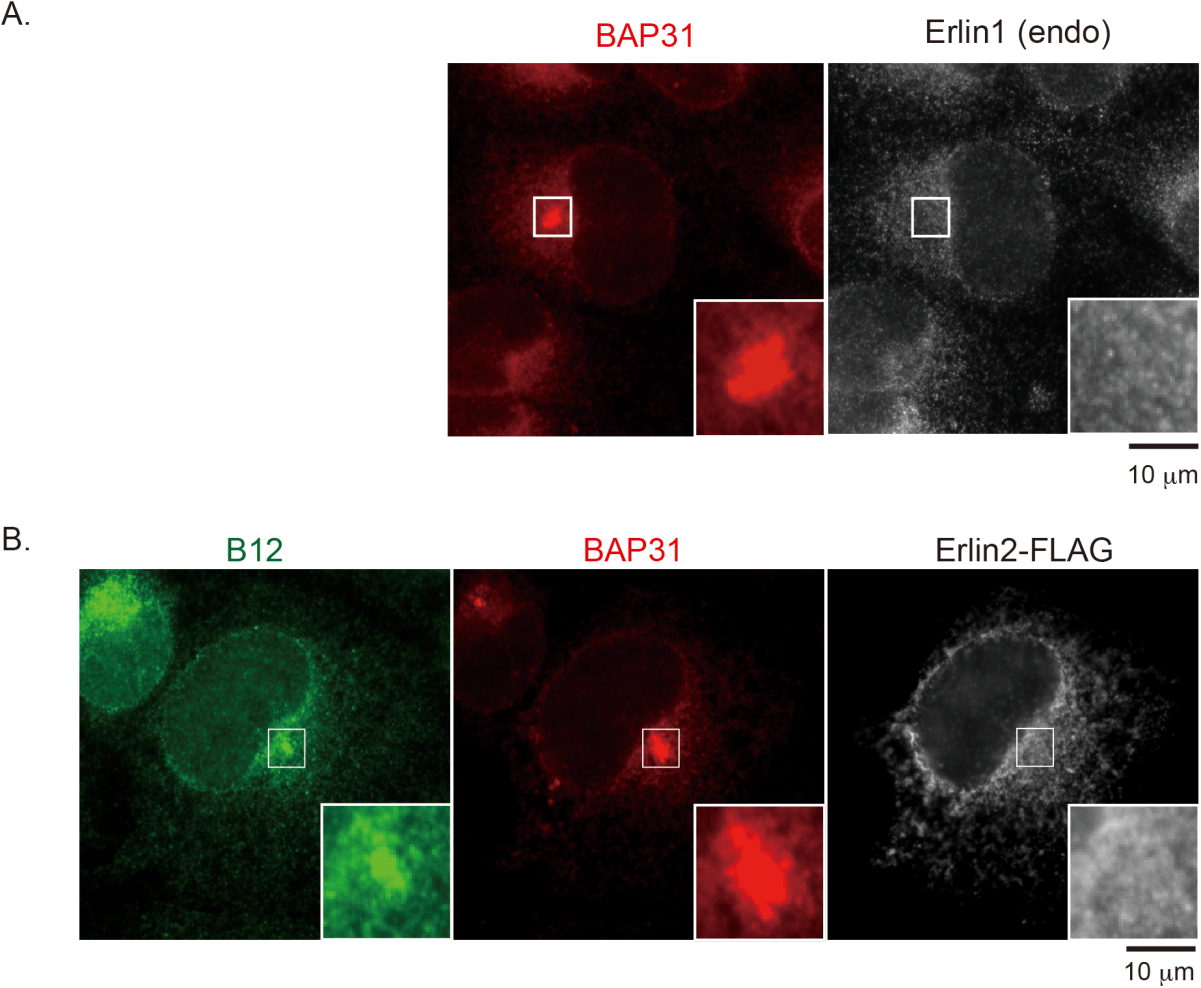
Erlin1 and Erlin2 do not reorganize into the SV40-induced foci structures. (A) CV-1 cells were infected with SV40 for 16 h, fixed, and subjected to immunofluorescence analysis using antibodies against BAP31 and Erlin1. Images were taken by epifluorescence microscopy. Inset shows a 3x enlarged image corresponding to the enclosed white square. Bar represents 10 μm. (B) CV-1 cells transfected with Erlin2*-FLAG were infected with SV40 for 16 h and analyzed as in A, except that antibodies against B12, BAP31, and FLAG were used.

The observation that B12’s association with Erlin1/2 is critical for B12 to reorganize into the foci raises the possibility that Erlin1/2 might also mobilize into the foci. However, we found that neither endogenous Erlin1 (Fig 5A) nor transfected Erlin2*-FLAG (Fig 5B) mobilized into the BAP31-positive foci during SV40 infection by epifluorescence microscopy. Confocal microscopy analyses similarly revealed that neither transfected Erlin1*-FLAG nor Erlin2*-FLAG reorganize into the SV40-induced BAP31-positive foci (S2 Fig). These results demonstrate that, despite the importance of the B12-Erlin1/2 interaction in supporting B12’s mobilization into the foci, Erlin1/2 themselves do not reorganize into these structures.

### SV40 induces the release of B12 from Erlin1 and Erlin2

**Fig 6:**
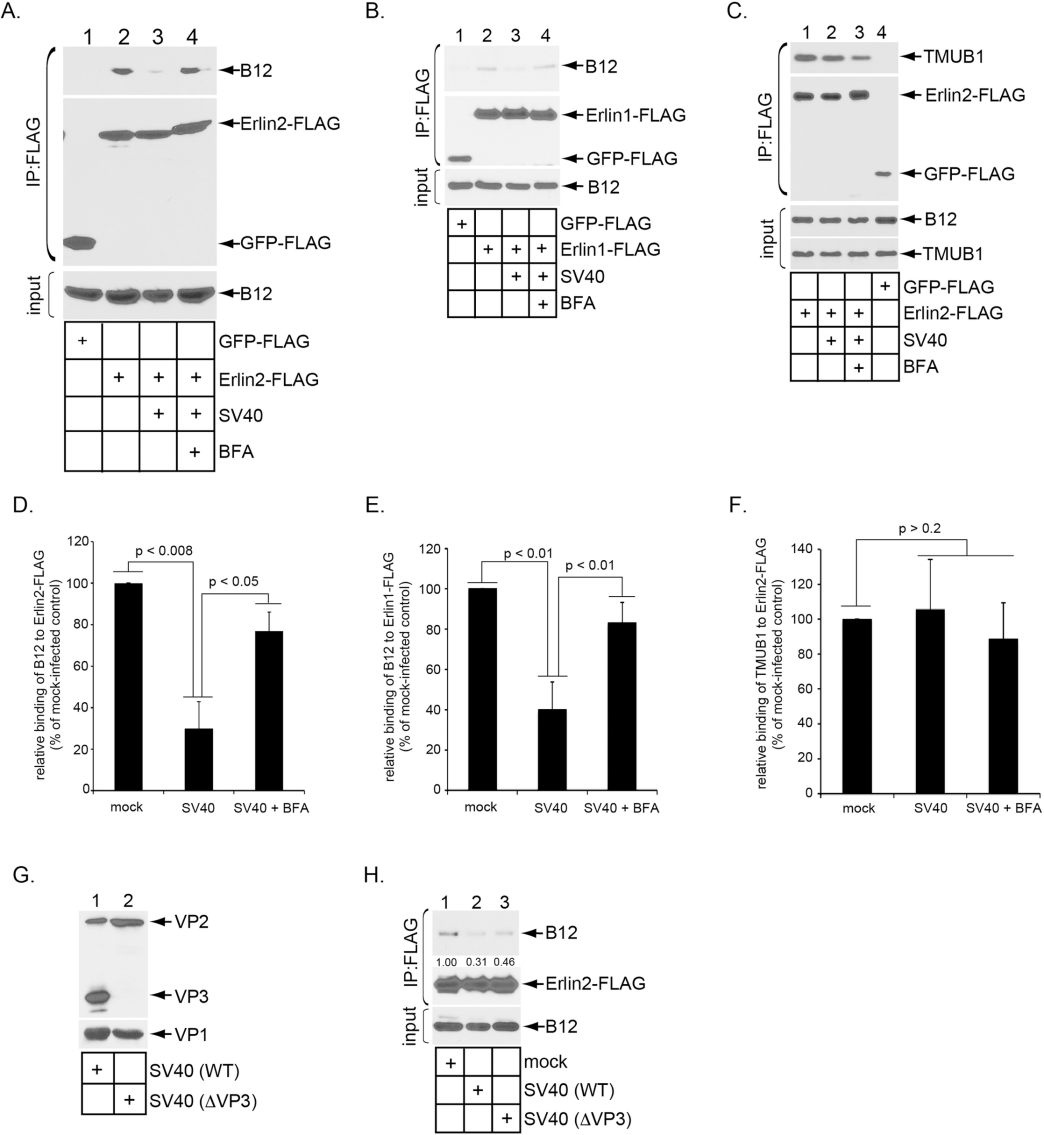
SV40 induces the release of B12 from Erlin1 and Erlin2. (A) CV-1 cells transfected with GFP-FLAG or Erlin2-FLAG were mock-infected or infected with SV40 for 16 h in the absence or presence of BFA. Cells were then harvested and lysed. The resulting whole cell lysates were subjected to immunoprecipitation using FLAG antibody-conjugated agarose beads and the immunoprecipitated samples analyzed by immunoblotting with the indicated antibodies. (B) As in A, except that Erlin1-FLAG was used. (C) As in A, except that an antibody against TMUB1 was used for immunoblotting. (D) The immunoprecipitated B12 band intensity in A was quantified using ImageJ (NIH). Data were normalized against mock-infected cells expressing Erlin2-FLAG, and represent the means ± standard deviations of data from at least 3 independent experiments. Student two-tailed *t* test was used to determine statistical significance. (E) As in D, except the immunoprecipitated B12 band intensity in B was quantified. (F) As in D, except the immunoprecipitated TMUB1 band intensity in C was quantified. (G) SV40 (WT) and SV40 (ΔVP3) were analyzed with SDS-PAGE, followed by immunoblotting with antibodies against VP1 and VP3. (H) As in A, except that CV-1 cells transfected with Erlin2-FLAG were mock-infected, infected with SV40 (WT), or infected with SV40 (ΔVP3) for 16 h. The immunoprecipitated B12 band intensity in A was quantified using ImageJ (NIH) and shown below the B12 band.

As B12 reorganizes into the foci during SV40 infection, we reasoned that this J-protein must move laterally along the ER lipid bilayer to reach the foci during virus infection. Because Erlin1/2 do not reorganize into the foci, we hypothesize that B12’s mobilization is initiated when it is released from Erlin1/2. To test this, we used a co-immunoprecipitation approach. Cells transfected with GFP-FLAG or Erlin2*-FLAG were mock-infected or infected with SV40. The resulting whole cell extracts were subjected to immunoprecipitation using FLAG antibody-conjugated agarose beads, and the precipitated proteins analyzed by SDS-PAGE and immunoblotting. We found that Erlin2*-FLAG but not GFP-FLAG co-precipitated endogenous B12 in mock-infected cells (Fig 6A, top panel, compare lane 2 to 1), as expected (Fig 1). However, SV40 infection significantly reduced the B12-Erlin2*-FLAG interaction (Fig 6A, top panel, compare lane 3 to 2; quantified in Fig 6D), suggesting that the virus triggers B12 release from Erlin2. SV40’s ability to reduce this interaction requires its presence in the ER because blocking virus trafficking to the ER (by incubating cells with BFA) prevented SV40 from releasing B12 from Erlin2 (Fig 6A, top panel, compare lane 4 to 3; quantified in Fig 6D). SV40 regulates the B12-Erlin1-FLAG interaction in a similar manner (Fig 6B; quantified in Fig 6E). Virus-induced release of B12 from Erlin1/2 is specific because SV40 does not affect binding of Erlin2*-FLAG to TMUB1 (Fig 6C, top panel, compare lane 2 to 1; quantified in Fig 6F), an established ER membrane partner of Erlin2 [33]. Our binding analyses thus demonstrate that SV40 specifically promotes release of B12 from Erlin1/2, likely to initiate B12’s mobilization and recruitment to the foci where it supports virus ER membrane penetration.

We previously found that an SV40 mutant lacking VP3 (SV40 (ΔVP3), but not VP2, can traffic from the cell surface to the ER [32], although this mutant virus cannot induce foci formation [4] nor penetrate the ER membrane [32]. To test if SV40 (ΔVP3) induces release of B12 from Erlin2, cells transfected with Erlin2*-FLAG were mock-infected, infected with WT SV40, or infected with SV40 (ΔVP3) (Fig 6G). Cells were then harvested 16 hpi, and the resulting whole cell lysates subjected to immunoprecipitation using FLAG antibody-conjugated agarose beads. We found that while WT SV40 markedly induced dissociation of endogenous B12 from Erlin2-FLAG, SV40 (ΔVP3) did so more modestly (Fig 6H, top panel, compare lanes 2 and 3 to 1), raising the possibility that VP3 may be involved in triggering B12 release from Erlin2.

### SV40-induced B12 release from Erlin2 is required for B12 mobilization to the foci leading to infection

**Fig 7:**
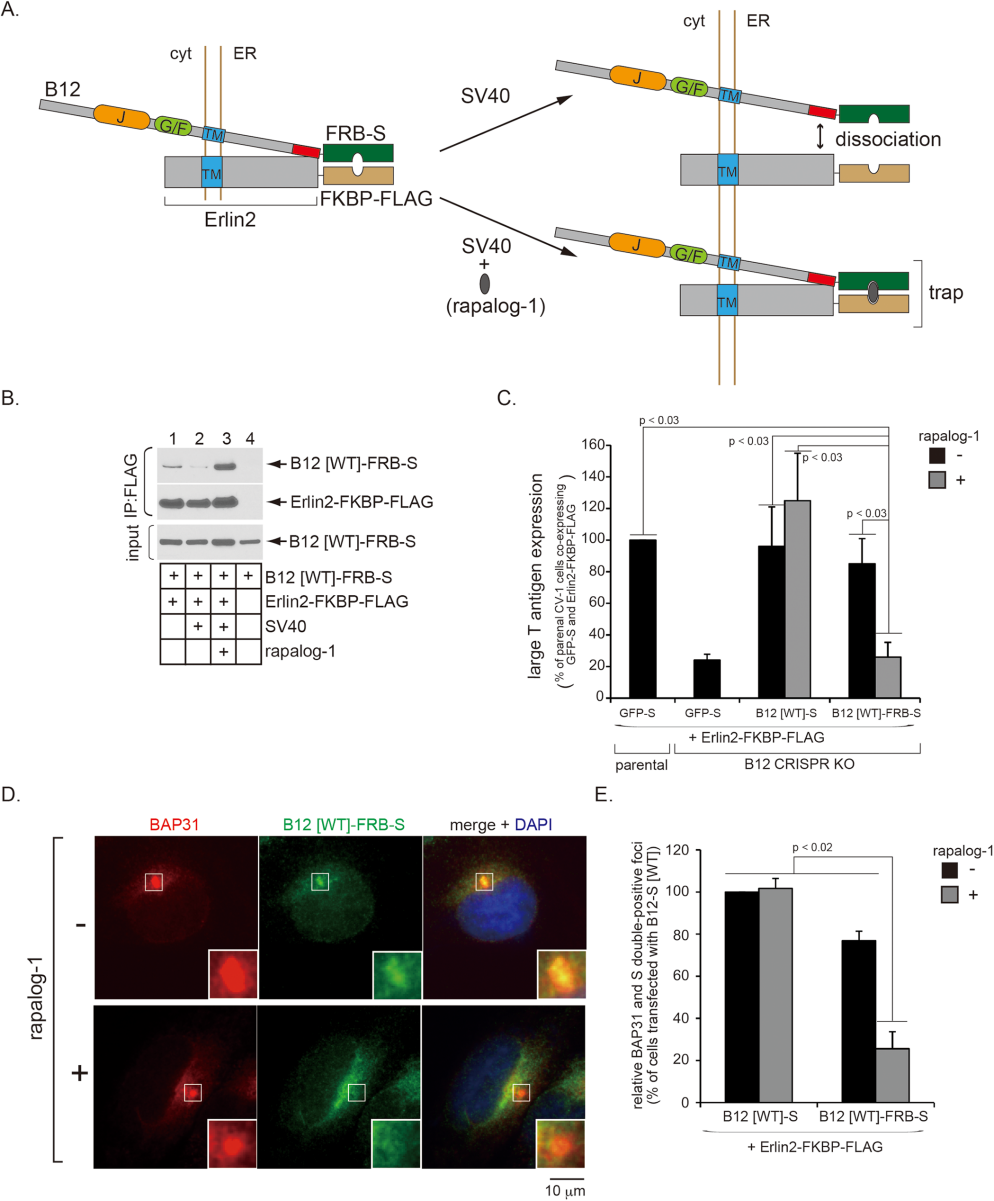
Trapping B12 to Erlin2 prevents B12 from mobilizing into SV40-induced foci and blocks infection. (A) A schematic describing the FKBP-FRB dimerization system used in this study. B12’s last 20 amino acids from the C-terminus that interact with Erlin1/2 are indicated in red. Although B12-FRB-S binds to Erlin2-FKBP-FLAG, B12-FRB-S is expected to dissociate from Erlin2-FKBP-FLAG during SV40 infection in the absence of raplog-1. However, in the presence of raplog-1 during SV40 infection, B12-FRB-S is trapped with Erlin2-FKBP-FLAG due to FKBP-FRB dimerization. (B) B12 CRISPR KO cells transfected with B12 [WT]-FRB-S with or without Erlin2-FKBP-FLAG were infected with SV40 or mock-infected for 6 h, and further incubated in the presence or absence of rapalog-1 for 10 h. Cells were harvested, lysed, and the resulting cell lysates subjected to immunoprecipitation using FLAG antibody-conjugated agarose beads. The immunoprecipitated samples were analyzed by immunoblotting with the indicated antibodies. (C) Parental and B12 CRISPR KO cells transfected with a vector co-expressing Erlin2-FKBP-FLAG and either GFP-S, B12 [WT]-S, or B12 [WT]-FRB-S were infected with SV40 for 6 h, further incubated in the absence or presence of rapalog-1 for 18 h, and subjected to immunofluorescence analysis using antibodies against S-tag and large T antigen. S-tag and large T antigen double-positive cells were counted and analyzed as in Fig 2C. Data are normalized against parental cells co-expressing Erlin2-FKBP-FLAG and GFP-S in the absence of rapalog-1, and represent the means ± standard deviations of data from at least 3 independent experiments. Student two-tailed *t* test was used to determine statistical significance. (D) B12 CRISPR KO cells transfected with a vector co-expressing Erlin2-FKBP-FLAG and B12 [WT]-FRB-S were infected with SV40 for 6 h, further incubated in the absence or presence of rapalog-1 for 10 h, and subjected to immunofluorescence analysis using antibodies against S-tag and BAP31. Images were taken by epifluorescence microscopy. Inset shows a 3x enlarged image corresponding to the enclosed white box. Bar represents 10 μm. (E) Numbers of cells with at least one S-tag and BAP31 double-positive focus in SV40-infected cells co-expressing Erlin2-FKBP-FLAG and either B12 [WT]-S or B12 [WT]-FRB-S in the absence or presence of rapalog-1 were counted. Data are normalized against cells co-expressing Erlin2-FKBP-FLAG and B12 [WT]-S in the absence of rapalog-1, and represent the means ± standard deviations of data from at least 3 independent experiments. Student two-tailed *t* test was used to determine statistical significance.

To unambiguously demonstrate that B12 release from the Erlins is necessary for B12 to reorganize into the foci to support SV40 infection, we employed a chemical-induced dimerization strategy to trap the B12-Erlin interaction in the presence of SV40. As depicted in Fig 7A, we generated a WT B12 construct containing an FRB domain and an S-tag at its C-terminus (B12 [WT]-FRB-S), and an Erlin2 construct containing an FKBP domain and a FLAG-tag at its C-terminus (Erlin2-FKBP-FLAG). As the rapamycin-1 analogue (rapalog-1) dimerizes FRB and FKBP [34], this drug should induce dimerization of B12 [WT]-FRB-S and Erlin2-FKBP-FLAG, thereby forcibly trapping B12 to Erlin2. To verify the integrity of this system, B12 CRISPR KO cells transfected with B12 [WT]-FRB-S with or without Erlin2-FKBP-FLAG were infected with SV40 or mock-infected in the absence or presence of rapalog-1. The resulting cell lysates were subjected to immunoprecipitation using FLAG antibody-conjugated agarose beads. Consistent with the endogenous B12-Erlin2-FLAG interaction (Fig 6A), precipitating Erlin2-FKBP-FLAG specifically pulled down B12 [WT]-FRB-S (Fig 7B, top panel, compare lane 1 to 4). SV40 infection decreased the B12 [WT]-FRB-S- Erlin2-FKBP-FLAG interaction (Fig 7B, top panel, compare lane 2 to 1). Importantly, adding rapalog-1 prevented the virus-induced decrease in interaction between these two proteins and appeared to have strengthened it (Fig 7B, top panel, compare lane 3 to 1 and 2). These results demonstrate that addition of rapalog-1 indeed induced hetero-dimerization of B12 [WT]-FRB-S and Erlin2-FKBP-FLAG, as described in Fig 7A.

We reasoned that if SV40-dependent B12 release from Erlin2 is required for B12’s reorganization to the foci to support virus ER membrane penetration leading to infection, artificially force-trapping B12 to Erlin2 should prevent B12 from mobilizing into the foci and thus preclude successful infection. To test this, B12 CRISPR KO and its parental CV-1 cells were transfected with Erlin2-FKBP-FLAG and either the control GFP-S, B12 [WT]-S, or B12 [WT]-FRB-S construct, and the experiments conducted with or without adding rapalog-1. Only cells expressing the S-tagged construct was scored for the expression of large T antigen. As expected (Fig 4F), B12 knockout significantly blocked SV40 infection (Fig 7C, compare second to first bar). Importantly, although expressing B12 [WT]-S completely restored infection regardless of the presence of rapalog-1 (Fig 7C, compare third and fourth to second bar), expressing B12 [WT]-FRB-S can only restore infection in rapalog-1’s absence (Fig 7C, compare fifth to sixth bar). Thus, an exogenously expressed B12 cannot rescue SV40 infection in B12 knockout cells if the B12 protein is force-trapped to Erlin2.

These results predict that exogenously expressed B12 [WT]-FRB-S, in the presence of the Erlin2-FKBP-FLAG binding partner, would be restricted from mobilizing into the foci upon addition of rapalog-1 during SV40 infection. Indeed, B12 [WT]-FRB-S can largely mobilize to the BAP31-positive foci during SV40 infection in the absence of rapalog-1, similar to B12 [WT]-S (Fig 7D and S3 Fig, compare first rows in both Figs; quantified in Fig 7E, compare third to first bar). By contrast, whereas B12 [WT]-S can reorganize into the virus-induced foci even in the presence of rapalog-1 (S3 Fig, second row; quantified in Fig 7E, second bar), B12 [WT]-FRB-S inefficiently mobilizes into this structure in the presence of rapalog-1 (Fig 7D, second row; quantified in Fig 7E, fourth bar). Hence, artificially entrapping B12 to Erlin2 prevents B12 from moving into the foci structure. These data are in agreement with the infection experiments, further supporting the hypothesis that SV40-induced release of B12 into the foci is essential for promoting virus ER membrane penetration leading to successful infection.

### The Erlin1 and Erlin2 complex anchors B12 in the ER

**Fig 8:**
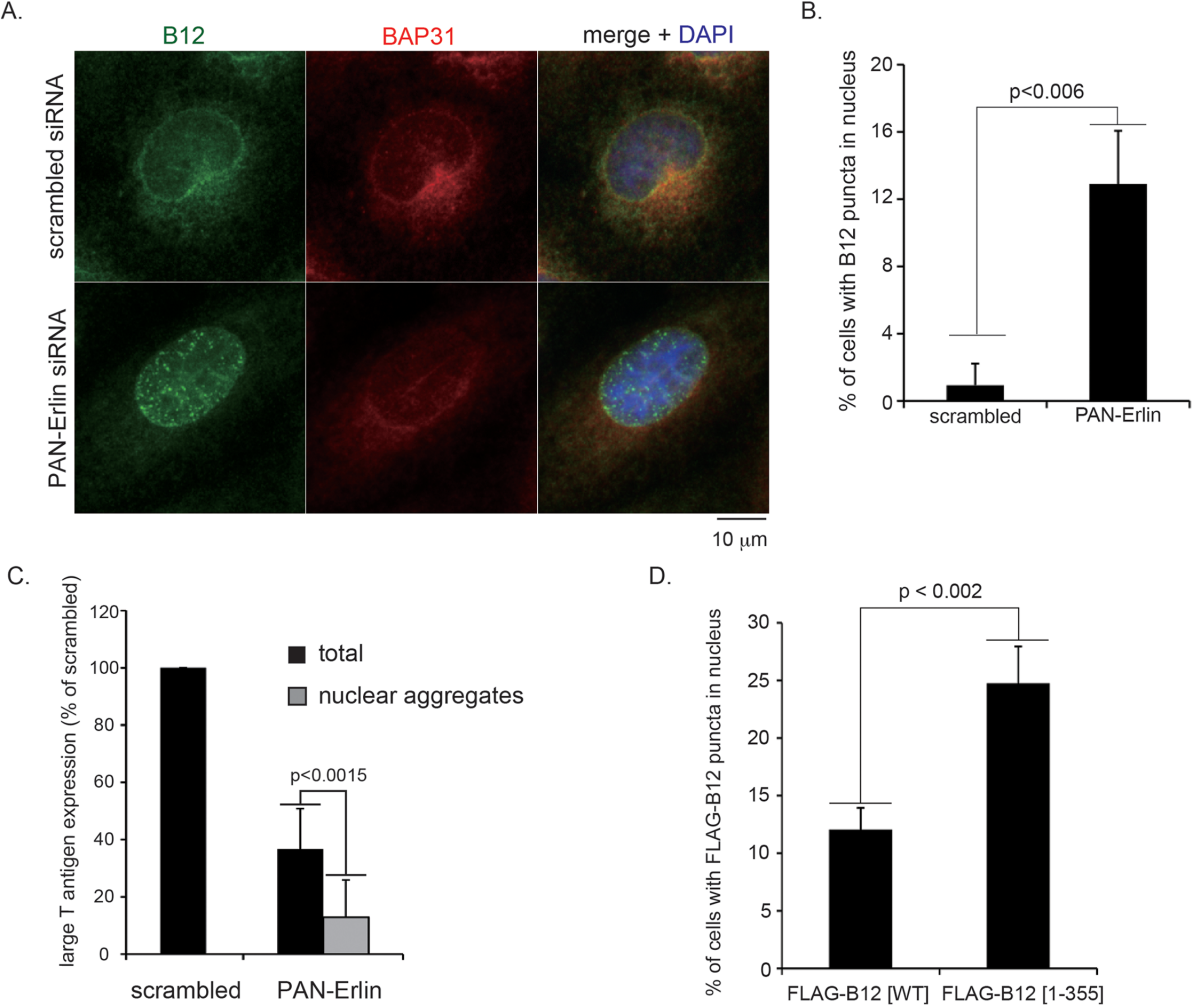
The Erlin1 and Erlin2 complex anchors B12 in the ER. (A) CV-1 cells transfected with scrambled or PAN-Erlin siRNA were fixed and subjected to immunofluorescence analysis using antibodies against B12 and BAP31. Images were taken by epifluorescence microscopy. Bar represents 10 μm. (B) Cells that have at least five B12-positive aggregates in the nuclei were scored. Data represent the means ± standard deviations of data from at least 3 independent experiments. Student two-tailed *t* test was used to determine statistical significance. (C) CV-1 cells transfected with scrambled or PAN-Erlin siRNA were infected with SV40, fixed, and subjected to immunofluorescence analysis using antibodies against B12 and large T antigen. Large T antigen-positive nuclei were analyzed as Fig 2C, except that in cells transfected with PAN-siRNA, the number of large T antigen-positive nuclei in cells displaying at least five B12-positive aggregates were counted. At least 300 nuclei from scrambled and PAN Erlin siRNA-transfected cells (total) and at least 100 nuclei from PAN Erlin siRNA-transfected cells displaying B12-positive nuclear aggregates were counted. Data are normalized against cells transfected with scrambled siRNA, and represent the means ± standard deviations of data from at least 3 independent experiments. Student two-tailed *t* test was used to determine statistical significance. (D) Cells transfected with truncated CMV-driven FLAG-B12 [WT] or FLAG-B12 [1–355] were fixed and subjected to immunofluorescence analysis using antibodies against FLAG. Cells with at least five B12-positive aggregates in the nuclei were scored as in A. Data represent the means ± standard deviations of data from at least 3 independent experiments. Student two-tailed *t* test was used to determine statistical significance.

Because B12’s release from Erlin1/2 is critical during SV40 ER membrane transport, we hypothesize that part of Erlin1/2’s function is to position B12 in the ER so that B12 can be properly released upon SV40 infection. Accordingly, we probed the fate of endogenous B12 in the absence of Erlin1/2 by epifluorescence microscopy and found that a fraction of B12 mislocalizes to small aggregates that co-localize with the nucleus (Fig 8A, compare top and bottom panels; quantified in Fig 8B). These small B12 aggregates were not artifacts caused by epifluorescence microscopy because confocal microscopy analyses demonstrated that B12 does not normally form such small aggregates (S4 Fig). These findings suggest that Erlin1/2 normally anchor B12 in the ER. In fact, when SV40 infection was evaluated in PAN-Erlin siRNA-transfected cells where B12 displays an obvious nuclear aggregation phenotype (as oppose to total cells transfected with the PAN-Erlin siRNA), the infection block was more severe (Fig 8C). Thus, when Erlin1/2 depletion can lead to mislocalization of B12 to the nucleus, SV40 infection is severely compromised.

The idea that B12 requires binding partners to anchor it in the ER is supported by a previous report demonstrating that overexpressed WT B12 localizes to the nucleus [35], presumably because the overexpressed B12 lacks appropriate levels of corresponding binding partners such as Erlin1/2 that would normally restrict it in the ER. To further support this view, we used a truncated CMV promoter that retains a very low transcriptional activity [36] to drive the expression of either FLAG-B12 [WT] or FLAG-B12 [1–355]. Our results revealed that FLAG-B12 [1–355] displayed an increased mislocalization to the nucleus when compared to FLAG-B12 [WT] (Fig 8D), likely because FLAG-B12 [1–355] is inefficiently anchored in the ER since it cannot bind to Erlin1/2 (Fig 4).

## Discussion

Membrane penetration by non-enveloped viruses remains an enigmatic process. One key question is whether the viral particle hijacks pre-existing protein-conducting channels embedded in the limiting membrane that would support the transport process, or if the virus creates de-novo membrane transport structures that facilitate membrane translocation. In the case of the non-enveloped SV40, where translocation across the ER membrane represents the decisive infection step, increasing evidence suggest that this virus does not exploit a pre-existing channel such as the ER membrane-bound E3 ubiquitin ligase Hrd1 [5,37,38]. Hrd1 is the central component of the retro-translocation channel that translocates misfolded ER proteins to the cytosol for proteasomal degradation in a pathway called ER-associated degradation (ERAD; [39]). Instead, SV40 induces reorganization of select ER membrane components into distinct puncta in the ER membrane called foci that are postulated to serve as the cytosol entry sites [4–6,21]. This represents the only example of a non-enveloped virus constructing its own membrane transport portal during entry.

**Fig 9:**
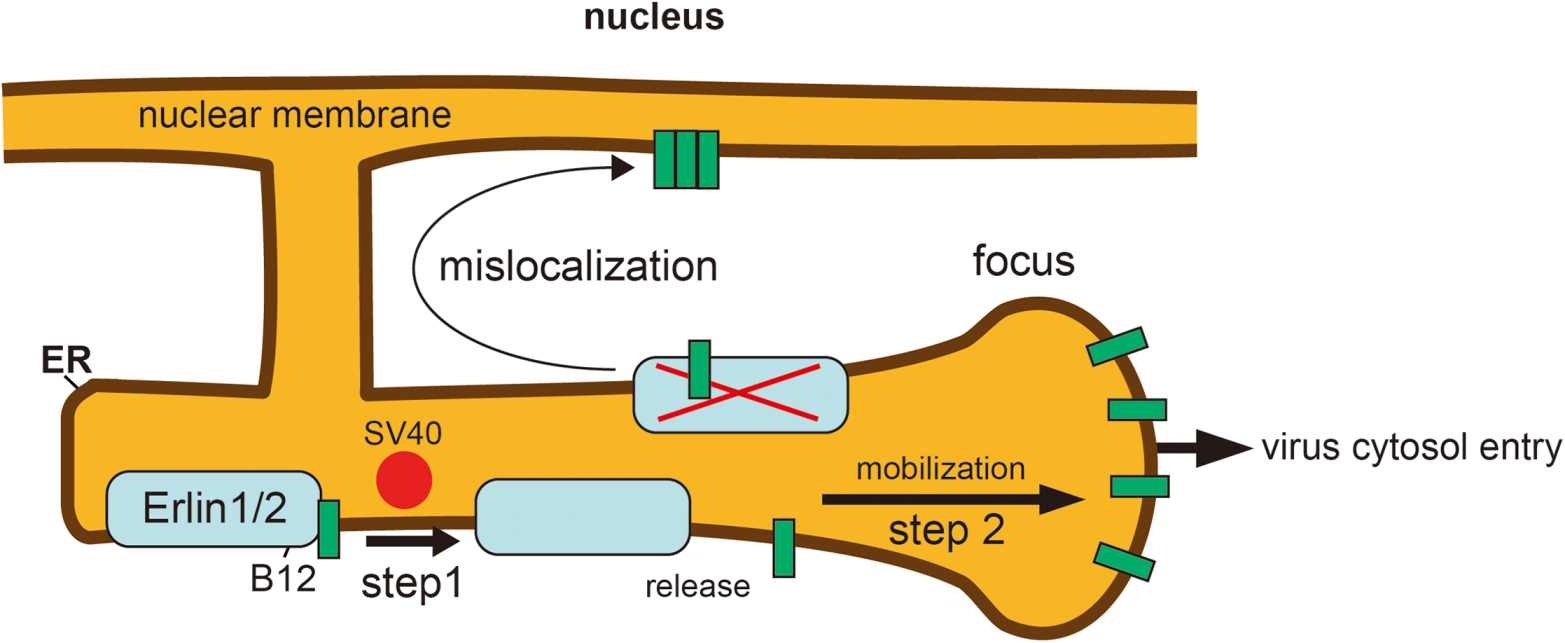
Model depicting the role of the Erlin1/2-B12 interaction during SV40 ER membrane penetration. The Erlin1 and Erlin2 (Erlin1/2) complex is identified as a binding partner of B12. When SV40 traffics from the cell surface to the ER, it triggers the release of B12 from the Erlin1/2 complex (step 1). The released B12 in turn mobilizes to the foci (step 2) where B12 promotes ER-to-cytosol membrane penetration of SV40 by recruiting a cytosolic extraction complex [6,21] that ejects the virus into the cytosol. In the absence of the Erlin1/2 complex, B12 can mislocalize to the nucleus, suggesting that Erlin1/2 acts to anchor B12 in the ER.

The J-protein B12 is one of the ER membrane proteins that mobilizes into the foci during SV40 infection [6]. In the foci, B12 assists in the recruitment of a cytosolic chaperone complex that ejects the virus into the cytosol in coordination with the two other transmembrane J-proteins B14 and C18 [4,6,21]. However, the molecular basis by which B12 mobilizes into the foci and the host factors co-opted to regulate this process remain unknown. In this study, we identified two closely related ER membrane proteins Erlin1 and Erlin2 (Erlin1/2) as cellular components that bind to B12 and facilitate B12’s reorganization into the SV40-induced foci. As proposed in a model depicted in Fig 9, we envision that Erlin1/2, which normally hetero-oligomerize, restrict B12 in the ER membrane by associating with B12. Upon arrival of SV40 to the ER lumen from the cell surface, it induces the release of B12 from the Erlin1/2 complex (step 1). The disengaged B12 in turn reorganizes into the foci in order to support ER-to-cytosol transport of SV40 (step 2). At present, how SV40 triggers B12’s release from Erlins is unclear. It is possible that SV40 directly interacts with and induces conformational changes to either B12 or Erlin1/2, causing these components to lose affinity for each other. Alternatively, during SV40 infection, B12 might attract other ER or cytosolic factors that competitively interfere with binding to the Erlin proteins. Future experiments are needed to clarify this important point. Regardless of the mechanism, the release step is required for B12’s mobilization into the foci where it promotes virus infection as artificially locking B12 to Erlin2 prevents these downstream events from occurring.

When Erlin1/2 is depleted, we found that endogenous B12 can mislocalize to the nucleus (Fig 9), suggesting that Erlins act as anchors, restricting B12 in the ER. Because Erlin1/2 have been shown to hetero-dimerize and form massive megadalton protein complexes [26–28], this intrinsic property may enable them to function as efficient anchoring factors. Consistent with this, another group demonstrated that overexpressed WT B12 similarly mislocalizes to the nucleus and forms nuclear globular structures termed DJANGOS [35], presumably due to insufficient levels of Erlins that are required to restrict the overexpressed proteins in the ER. The observation that an ER membrane protein such as B12 can localize incorrectly to the nucleus is not surprising since the ER membrane is topologically contiguous with the outer nuclear membrane. B14 and C18 also promote SV40 ER membrane penetration and infection [20,22]. Intriguingly, both of these proteins reorganize into the foci in a virus-dependent manner [4,6]. Because B14 does not bind to Erlin1/2, the mechanism by which it is recruited into the foci must be different than B12. However, as we previously demonstrated that C18 engages Erlin1/2 [20], C18’s mechanism of release/recruitment into the foci might be similar to B12.

Erlin1/2-dependent restriction of B12 in the ER is highly reminiscent of the mechanism by which Erlin1/2 restricts the ER membrane protein sterol-regulated element binding protein (SREBP) in the ER through a direct interaction with the SREBP-SCAP complex [40]. SREBP is normally kept in the ER but must mobilize to the Golgi when the cholesterol level is low. In the Golgi, SREBP is cleaved to generate an active transcription factor, which in turn moves to the nucleus to upregulate genes responsible for cholesterol synthesis [41]. The parallel finding that Erlins anchor yet another transmembrane protein in the ER suggests that Erlins might function as a general ER-anchoring factor. Moreover, given that Erlins have been shown to play a poorly-defined role in ERAD [39], the possibility that Erlins might act to restrict various ERAD membrane components in the ER should now be explored.

## Materials and methods

Polyclonal Hsp90 and monoclonal SV40 large T antigen antibodies were purchased from Santa Cruz Biotechnology (Santa Cruz, CA), a monoclonal PDI antibody from Abcam (Cambridge, MA), a monoclonal BiP antibody from BD (Franklin, NJ), a monoclonal FLAG antibody from Sigma-Aldrich (St. Louis, MO), polyclonal Erlin1, B12, B14, and TMUB1 antibodies from Protein Tech Group (Chicago, Il), a polyclonal Hsc70 antibody and a monoclonal BAP31 antibody (CC-1) from Thermo Fisher Scientific (Waltham, MA), and a polyclonal Erlin2 antibody from Cell Signaling Technology (Danvers, MA). Monoclonal antibodies against SV40 VP1 were generous gift from Dr. W. Scott (University of Miami), and polyclonal antibodies against SV40 VP3 were from Dr. A. Helenius (ETH Zurich, Switzerland). FLAG M2 antibody-conjugated agarose beads, 3xFLAG peptide, Triton X-100 and Brefeldin A (BFA) were purchased from Sigma-Aldrich, PMSF, digitonin, and Deoxy Big Chap from EMD Millipore Chemicals (San Diego, CA), Protein G magnetic beads, Lipofectamine 2000, Lipofectamine RNAi max, and Hygromycin B from Thermo Fisher Scientific, Fugene HD from Promega (Madison, WI), Polyethylenimine HCl MAX, Linear, Mw 40,000 (PEI) from Polysciences Inc. (Warminster, PA), the rapamycin-1 anologue heterodimerizer from Takara Bio Inc. (Kusatsu, Shiga, JAPAN), and Blasticidin S HCl from Corning (Corning, NY). CV-1 and HEK 293T cells (ATCC, Manassas, VA) were cultured in DMEM (Thermo Fisher Scientific) containing 10% fetal bovine serum (Corning), 10 U/ml penicillin (Thermo fisher Scientific), and 10 μg/ml streptomycin (Thermo Fisher Scientific).

### DNA constructs

To construct a transfer vector in order to introduce 3xFLAG-B12 into Flp-in 293 T-REx cells, the 3xFLAG tag sequence was attached to the B12 cDNA by PCR using oligos containing the corresponding sequence and the resulting PCR product inserted into pcDNA5 FRT/TO. The human Erlin1 cDNA was amplified from the HEK 293T cDNA library and the human Erlin2 cDNA was a generous gift from Dr. R. DeBose-Boyd (University of Texas Southwestern Medical Center). To generate siRNA-resistant Erlin1 and Erlin2 constructs, the following silent mutations were introduced into each cDNA using an overlapping PCR method: Erlin1: 248 GGG-GTT-ATG-ATT-TAC-ATC-GAT-AGG 272, and Erlin2: 241 GGT-GTG-ATG-ATT-TAC-TTC-GAT-CGG 265, where the underlines denote the introduced silent mutation. The resulting PCR products were inserted into pCDNA3.1(-) in frame with the FLAG tag sequence by standard cloning methods. FLAG-B12 [WT] and FLAG-B12 [H138Q] were previously described [22,25] and FLAG-B12 [1–355] was constructed by amplifying the corresponding DNA sequence and sub-cloning it into pcDNA3.1 (-). For the S-tagged constructs used in this study, PCR-amplified cDNA sequences were inserted into pcDNA3.1(-) in frame with the S tag sequence by standard cloning methods. To express low level of B12 constructs, the enhancer region of the full length CMV promoter in pcDNA3.1 (-) (234–773) was deleted by an inverse PCR method [36]. To fuse FKBP to Erlin2-FLAG, a PCR-amplified FKBP sequence was introduced between the Erlin2 and FLAG-tag DNA sequences using standard cloning methods. Similarly, a PCR-amplified FRB sequence was introduced between the B12 and S-tag DNA sequences. To construct vectors co-expressing CMV-driven Erlin2-FKBP-FLAG and either truncated CMV-driven GFP-S, B12 [WT]-S, or B12 [WT]-FRB-S, an expression cassette containing the truncated CMV promoter, the S-tagged gene, and the polyA sequences (with BglII and MluI sites attached at 5’ and 3’ ends, respectively) was amplified by PCR and inserted into BglII- and MluI-digested pcDNA/Erlin2-FKBP-FLAG.

### Affinity purification of 3xFLAG B12

Flp-in 293 T-REx cell stably expressing 3xFLAG-B12 were harvested and semi-permeabilized in a buffer containing 50 mM Hepes (pH 7.5), 150 mM NaCl, 0.02% digitonin, and 1 mM PMSF at 4°C for 10 min. Following centrifugation at 16,100x g for 10 min at 4°C, the resulting pellet fraction was further lysed in a buffer containing 50 mM Hepes (pH 7.5), 150 mM NaCl, 1% deoxy Big CHAP and 1 mM PMSF at 4°C for 10 min and centrifuged at 16,100x g for 10 min at 4°C. The resulting supernatant fraction was further centrifuged at 50,000 rpm for 10 min at 4°C in a TLA-100.3 rotor (Beckman Coulter, Brea, CA) to remove residual detergent-insoluble materials. The cleared lysates were incubated with anti-FLAG M2 antibody for 2 h at 4°C, mixed with Protein G magnetic beads, and further incubated for 2 h at 4°C. After the magnetic beads were extensively washed with a buffer containing 50 mM Hepes (pH 7.5), 150 mM NaCl, and 0.1% deoxy Big CHAP, bound materials were eluted with 0.1 mg/ml 3xFLAG peptide or SDS sample buffer.

### SV40 preparation

SV40 was prepared as described previously in Inoue and Tsai, 2011 [32].

### SV40 ER-to-cytosol transport assay

24–48 h post siRNA transfection, CV-1 cells were incubated with SV40 (MOI ~20) at 4°C for 1 h, washed, and incubated at 37°C for 12 h. Cells were harvested with a scraper, semi-permeabilized with a buffer containing 50 mM Hepes (pH 7.5), 150 mM NaCl, 0.1% digitonin, and 1 mM PMSF at 4°C for 10 min, and centrifuged at 16,100x g for 10 min. The resulting supernatant is referred to as the ‘cytosol’ fraction, while the resulting pellet (resuspended in SDS sample buffer) is referred to as ‘membrane’ fraction. Where indicated, the membrane fraction was further treated with a buffer containing 50 mM Hepes (pH 7.5), 150 mM NaCl, 1% Triton X-100, and 1 mM PMSF at 4°C for 10 min, and centrifuged at 16,100x g for 10 min. This second supernatant is the Triton X-100-soluble membrane fraction harboring ER-localized SV40.

### B12-Erlin1/2 binding assays

Approximately 1x10^6^ HEK 293T cells were plated on a 6-cm plate, incubated for 24 h, and transfected with the indicated constructs using PEI. 24 h after DNA transfection, cells were harvested and lysed in a buffer containing 50 mM Hepes (pH 7.5), 150 mM NaCl, 1% Triton X-100, and 1 mM PMSF. Following centrifugation at 16,100x g for 10 min, the resulting supernatant fractions were incubated with FLAG antibody-conjugated agarose beads at 4°C for 2 h. After the beads were extensively washed with lysis buffer, bound proteins were eluted from the beads with SDS sample buffer and subjected to SDS-PAGE followed by immunoblotting with the indicated antibodies. To analyze SV40-induced B12 release from Erlin1/2, approximately 3x10^5^ CV-1 cells were plated on a 6-cm plate, incubated for 24 h, and transfected with the indicated DNA constructs using Fugene HD. 24 h after DNA transfection, cells were infected with SV40 (MOI~100) for 16 h in the absence or presence of BFA, harvested, and analyzed as above. The B12 [WT]-FRB-S-Erlin2-FKBP-FLAG interaction was analyzed similarly. Briefly, approximately 3x10^5^ CRISPR B12 KO CV-1 cells were plated on a 6-cm plate, incubated for 24 h, and transfected with the indicated DNA constructs using PEI. 24 h after DNA transfection, cells were infected with mock or SV40 (MOI~100) for 6 h and further incubated in the absence or presence of 0.5 μM rapalog-1 heterodimerizer for 10 h, harvested, and analyzed as above.

### siRNA-mediated gene knockdown

The target sequences of the siRNAs and their final concentrations used in this study are as follows: Erlin 1 siRNA: Thermo Fisher Predesigned Silencer siRNA, ID# 1473100 (50 μM), Erlin 2 siRNA: GAACUAUACUGCUGACUAU[dT][dT] (10 μM), PAN-Erlin siRNA: Thermo Fisher Predesigned Stealth RNAi siRNA, ID# HSS116356 (50 μM).

Allstar negative control siRNA (Qiagen, Hilden, Germany) was used as a scrambled control siRNA. CV-1 cells were reverse-transfected with each siRNA at the indicated concentration using Lipofectamine RNAi MAX and incubated for 24 h (Erlin1 and PAN-Erlin siRNAs) or 48 h (Erlin 2 siRNA) prior to SV40 infection.

### XBP1 splicing

Evaluation of XBP1 splicing was described previously in Ravindran et al., 2015 [21].

### SV40 infection assay

CV-1 cells transfected with siRNAs were infected with SV40 (MOI~0.3) for 24 h and fixed with 1% paraformaldehyde. Following permeabilization with 0.2% Triton X-100, fixed cells were stained with a mouse monoclonal SV40 large T antigen antibody, followed by Alexa Fluor 488-conjugated secondary antibodies. In each experiment, approximately 1,000 cells were counted to assess the extent of large T antigen expression. For the Erlin1/2 rescue experiments, cells were initially transfected with siRNA, incubated for 24 h, and further transfected with FLAG-tagged constructs using Fugene HD. 24 h post DNA transfection, cells were infected with SV40, fixed, and permeabilized as described above. Fixed cells were stained with a mouse monoclonal SV40 large T antigen antibody and a rabbit monoclonal FLAG antibody followed by Alexa Fluor 594-conjugated anti-mouse IgG and Alexa Fluor 488-conjugated anti-rabbit secondary antibodies. Large T antigen expression was evaluated in cells expressing the FLAG-tagged protein. At least 100 cells for each sample were counted to assess the extent of large T antigen expression. B12 rescue experiments were similarly performed, except that parental CV-1 or B12 CRISPR KO cells were used without siRNA transfection. As indicated in the result and discussion sections, overexpressed B12 often mislocalized in the nucleus. To avoid artifacts caused by B12 mislocaliztion, only cells expressing FLAG-B12 [WT] or [1–355] that display a clear ER-localization patterns were counted as “FLAG-B12 [WT] or [1–355]-expressing” cells, unless otherwise noted.

### SV40-induced foci formation assay

CV-1 cells transfected with the indicated siRNAs or DNA constructs were infected with SV40 (MOI~30–50) for the indicated time. Cells were fixed and subjected to an immunofluorescence-based method as described in the SV40 infection assay, except that a rat monoclonal BAP31 antibody, the indicated primary antibodies, Alexa Fluor 594-conjugated anti-mouse IgG, and Alexa Fluor 488-conjugated anti-rabbit IgG were used. For triple staining, a rat monoclonal BAP31 antibody, a rabbit polyclonal B12 antibody, and a monoclonal mouse FLAG antibody were used as primary antibodies, while Alexa Fluor 594-conjugated anti-rat IgG, Alexa Fluor 488-conjugated anti-rabbit IgG, and Alexa Fluo-350-conjugated anti-mouse IgG secondary antibodies were used as secondary antibodies.

### FKBP/FRB dimerization assays

Parental and B12 CRISPR KO CV-1 cells were plated on coverslips in a 24-well plate at a density of approximately 2.5x10^4^ cells/well, incubated for 24 h, and transfected with DNA constructs using Fugene HD. 24 h after DNA transfection, cells were infected with SV40 (MOI~0.3) for 6 h and further incubated in the absence or presence of 0.5 μM rapalog-1 heterodimerizer for 18 h. 24 hpi, cells were fixed and subjected to an immunofluorescence method using a mouse monoclonal SV40 large T antigen antibody and a rabbit polyclonal S-tag antibody as described above. Fixed cell samples were imaged using Nikon Eclipse TE2000-E equipped with a 40x objective and a 12-bit CCD camera. To avoid potential overexpression-induced artifacts, cells were counted and examined for the large T antigen expression when expression of B12 [WT]-S or B12 [WT]-FRB-S was localized in the ER without forming nuclear aggregates and that their signal values range from 200 to 1000 under 12-bit resolution. At least 100 cells were counted to assess the extent of large T antigen expression. For SV40-induced foci formation assay, cells were transfected in the same manner, infected with SV40 (MOI~30–50) for 6 h and further incubated in the absence or presence of 0.5 μM rapalog-1 heterodimerizer for 10 h. Following the immunofluorescence procedure using anti S-tag and BAP31 antibodies, cell images were taken as in the infection assay, except that instead of assessing the large T antigen expression, BAP31 images were captured. At least 100 cells were counted to assess the extent of BAP31 and S double-positive foci formation in each experiment.

### Evaluating mislocalization of exogenously-expressed FLAG-tagged B12 constructs

Cells transfected with truncated CMV promoter-driven FLAG-B12 [WT] or FLAG-B12 [1–355] were fixed, subjected to the above-mentioned immunofluorescence method using a rabbit monoclonal FLAG antibody and Alexa Fluor 488-conjugated anti-rabbit IgG, and the samples were imaged as described above. To compare the expression level of FLAG-B12 [WT] and FLAG-B12 [1–355], all images were taken using the same exposure time. Due to the varied expression level of FLAG-tagged proteins among cells, cells were counted and assessed for mislocalization of FLAG-tagged B12 constructs only when the signal values of the ER-localized FLAG-tagged B12 constructs ranged from 500 to 1500 under 12-bit resolution and/or the FLAG-tagged B12 constructs formed small nuclear aggregate structures. By contrast, cells were considered to have overexpressed exogenous B12 and removed from analysis when the signal values of ER-localized FLAG-tagged B12 constructs were above 1500 under 12-bit resolution and/or the FLAG-tagged B12 constructs formed massive nuclear aggregated structures. At least 100 cells were counted to assess mislocalization of FLAG-tagged B12 constructs in each experiment.

### Generating stable cell lines

Flp-In 293 T-REx cells (Thermo Fisher Sciences) were co-transfected with pOG44 (Thermo Fisher Sciences) and pcDNA5 FRT/TO encoding 3xFLAG-B12 [WT] using Lipofectamine 2000. 24 h post transfection, cells were split and cultured in DMEM medium containing 100 μg/ml hygromycin and 5 μg/ml blasticidin for 10–15 days. Hygromycin-resistant colonies were cloned.

### Generating CRISPR KO cells

PX330 and PX459 used for generating B12 KO cells were gifts from Dr. F. Zhang (Addgene plasmid # 42230 and 62988) [42]. The following two oligonucleotides containing the +325 to +344 sequence from the transcription start site of the human DNAJB12 (GI: 194306639) were annealed and inserted into PX459. Fw: CACCGAG GAAGCGGAGCG CCCGGTC. Rv: AAACGACCGGGCGCTCCGCTTCCTC. The PX459 encoding the guide RNA or control PX330 was transfected into CV-1 cells using Fugne HD and 24 h post transfection, the medium was replaced with fresh medium containing 6 μg/ml puromycin. After most control cells have died off, the surviving cells transfected with the PX459 construct were re-plated for single colony isolation. When single colonies were fully grown and became visible, they were isolated with cloning cylinders. One of the established cell lines was further grown, and the resulting whole cell lysate was subjected to immunoblotting using anti-B12 antibodies. The genome was also isolated from this cell line and served as a template to amplify a region corresponding to the +273 to +619 sequence from the transcriptional start site of the human DNAJB12 (GI: 194306639) using the PCR primers: Forward: CTCACACTCA CCGCGAACTC and Reverse: GCACCATTATTGACACCTAC. The amplified PCR product was subjected to agarose gel electrophoresis, and the corresponding band was excised from the gel and purified. The isolated product was analyzed by Sanger sequencing using the following primer: 5’-GACATACGGTAGCACTTCCAC-3’. Because only one sharp peak was observed by Sanger analysis in which an “A” insertion was detected between the +341 and +342 positions, we concluded that both alleles have the same mutation.

## Supporting information

S1 Fig Sequence of the mutated region in CV-1 B12 CRISPR KO cells. (TIF)

S2 Fig Confocal microscopy images demonstrating that Erlins do not reorganize into the SV40-induced foci. (A) CV-1 cells transfected with Erlin1*-FLAG were infected with SV40 for 16 h, fixed, and subjected to immunofluorescence analyses using antibodies against BAP31 and FLAG. Images were taken by confocal microscopy. Inset shows a 2.5x enlarged image corresponding to the enclosed white square. Bar represents 10 μm. (B) As in A, except that Erlin2*-FLAG was used.
(TIF)

S3 Fig SV40-induced foci formation in B12 CRISPR KO cells coexpressing Erlin2-FKBP-FLAG and B12 [WT]-S. As in Fig 7D, except that B12 CRISPR KO cells transfected with a vector co-expressing Erlin2-FKBP-FLAG and B12 [WT]-S were used. Bar represents 10 μm.
(TIF)

S4 Fig Confocal microscopy images of the ER membrane proteins B12 and BAP31. Cells transfected with scrambled siRNA were fixed and subjected to immunofluorescence analyses using antibodies against B12 and BAP31. Images were taken by confocal microscopy. Bar represents 10 μm.
(TIF)
