# Supplementary figures and images for: Regulated Erlin-dependent release of the B12 transmembrane J-protein promotes ER membrane penetration of a non-enveloped virus
Source: PLoS Pathog. 2017 Jun 14;13(6):e1006439. doi: 10.1371/journal.ppat.1006439 (PMC5484543; doi:10.1371/journal.ppat.1006439)

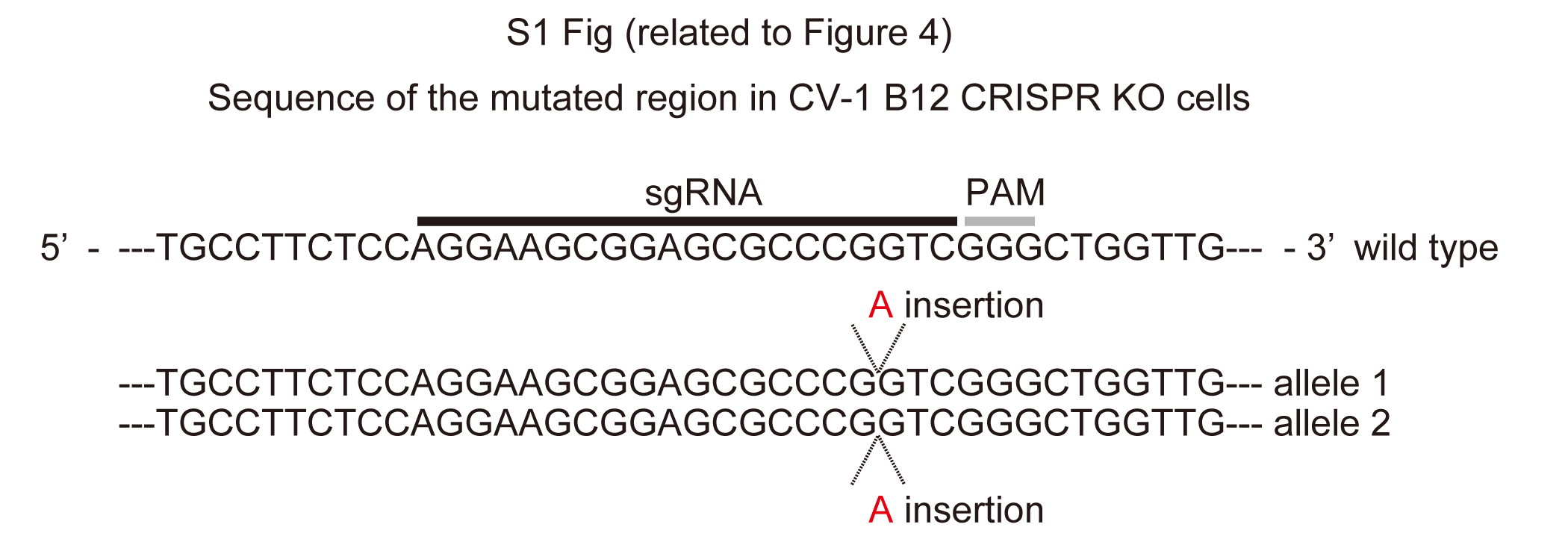

Supplement: S1 Fig — (TIF) [file ppat.1006439.s001.tif]

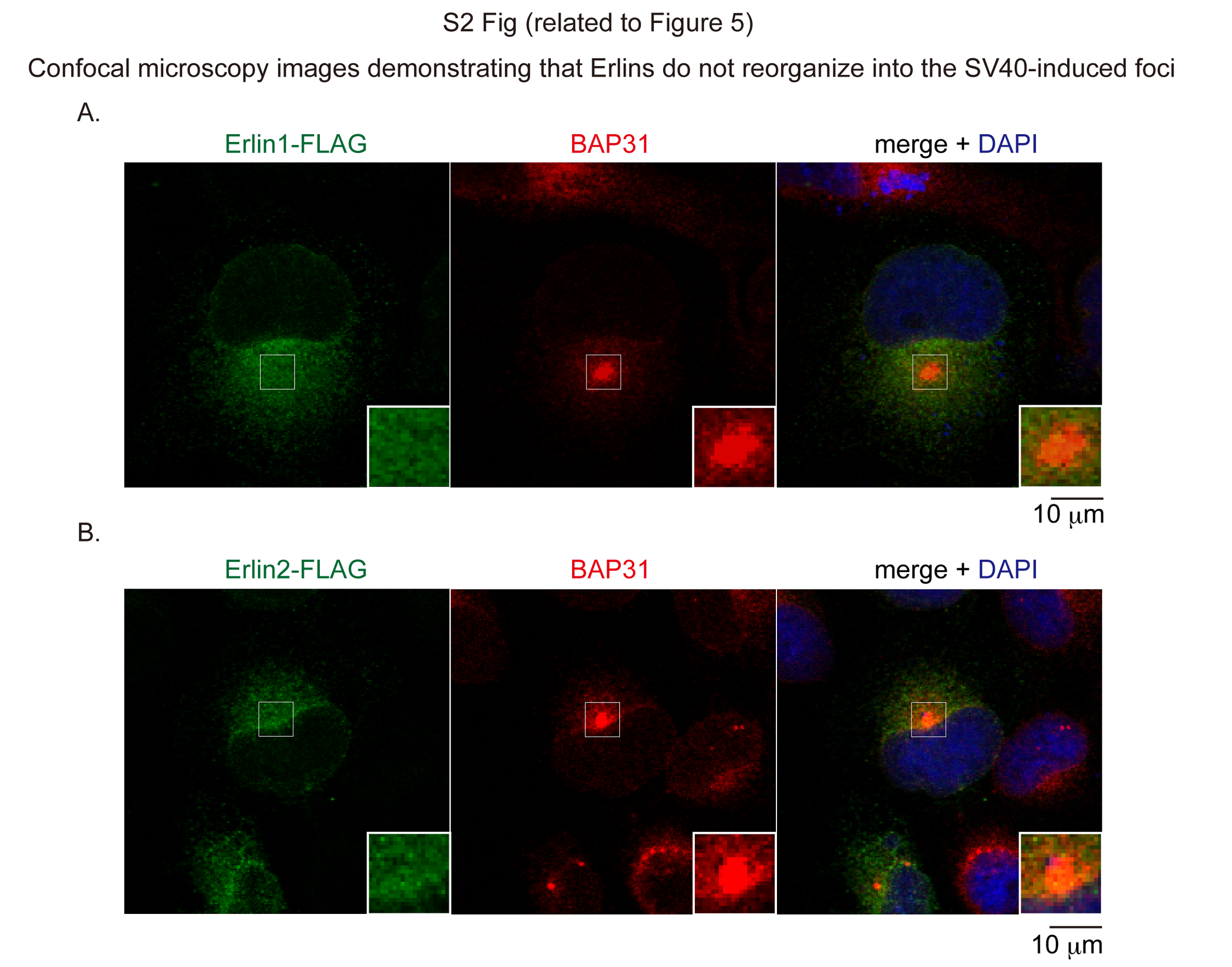

Supplement: S2 Fig — (A) CV-1 cells transfected with Erlin1*-FLAG were infected with SV40 for 16 h, fixed, and subjected to immunofluorescence analyses using antibodies against BAP31 and FLAG. Images were taken by confocal microscopy. Inset shows a 2.5x enlarged image corresponding to the enclosed white square. Bar represents 10 μm. (B) As in A, except that Erlin2*-FLAG was used. (TIF) [file ppat.1006439.s002.tif]

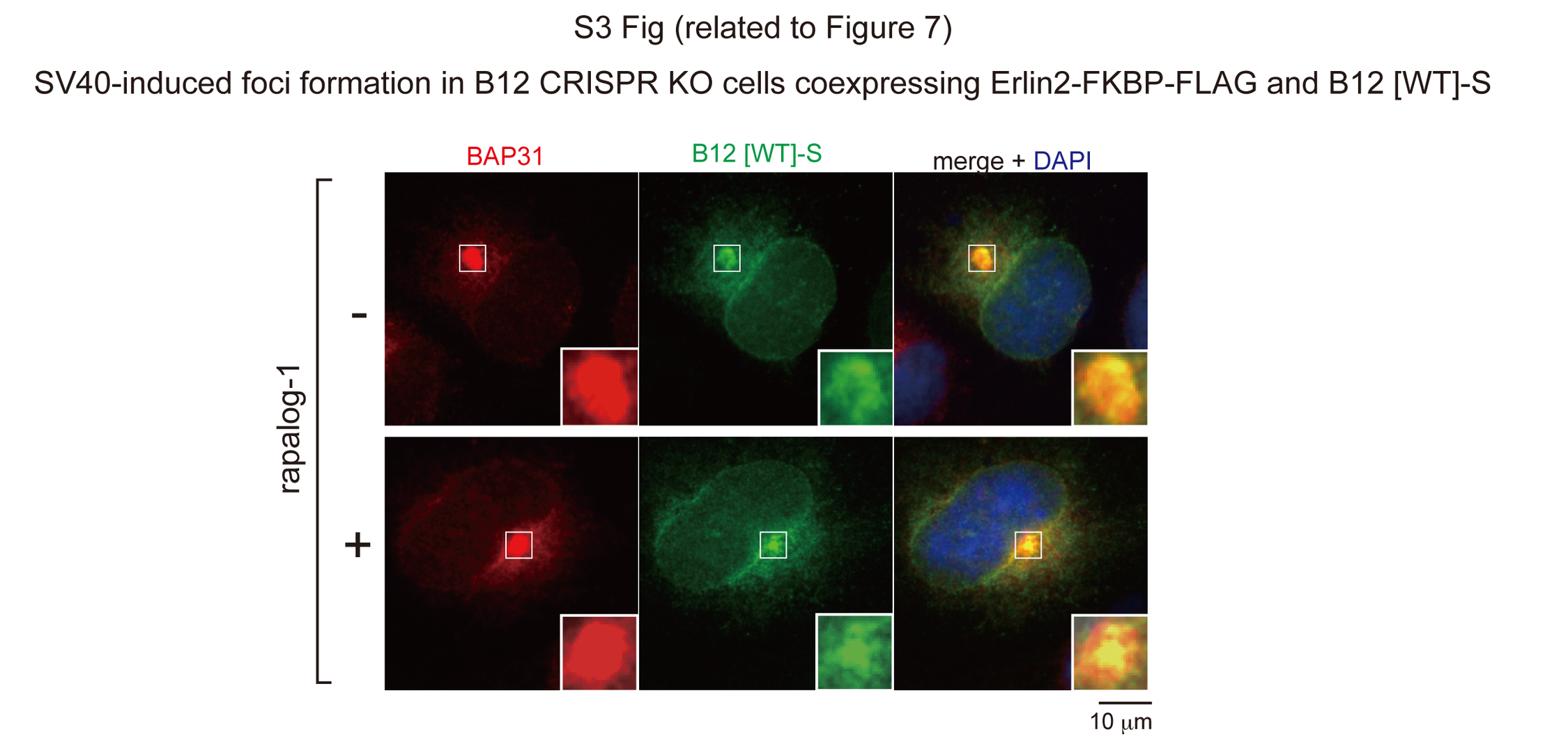

Supplement: S3 Fig — As in Fig 7D, except that B12 CRISPR KO cells transfected with a vector co-expressing Erlin2-FKBP-FLAG and B12 [WT]-S were used. Bar represents 10 μm. (TIF) [file ppat.1006439.s003.tif]

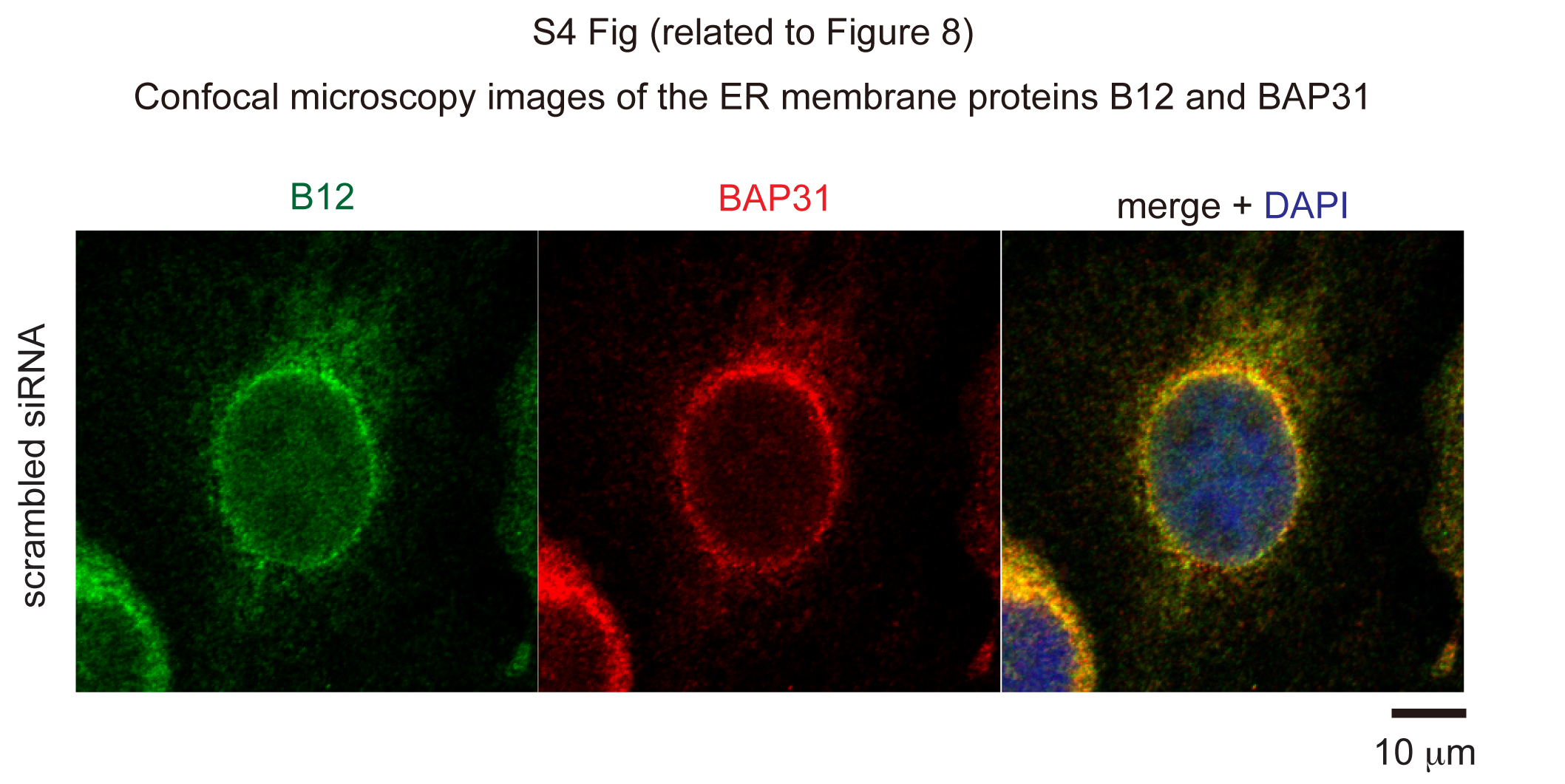

Supplement: S4 Fig — Cells transfected with scrambled siRNA were fixed and subjected to immunofluorescence analyses using antibodies against B12 and BAP31. Images were taken by confocal microscopy. Bar represents 10 μm. (TIF) [file ppat.1006439.s004.tif]
